# Reporting Quality of Randomized Controlled Trials for the Treatment of Eczema with Chinese Patent Medicine Based on the CONSORT-CHM Formulas 2017

**DOI:** 10.1155/2020/2949125

**Published:** 2020-09-14

**Authors:** Ming Li, Boyang Zhou, Lihong Zhou, Linfeng Li

**Affiliations:** Department of Dermatology, Beijing Friendship Hospital, Capital Medical University, 95 Yong'an Road, Xicheng District, Beijing 100050, China

## Abstract

**Objective:**

Chinese patent medicine (CPM) has been widely used to treat eczema in mainland China for decades. This study aims to investigate circulating CPM for eczema in mainland China and to evaluate the reporting quality of randomized controlled trials (RCTs) of them by using the CONSORT-CHM formulas 2017 (Consolidated Standards of Reporting Trials for Chinese herbal medicine formulas 2017).

**Methods:**

Circulating CPM with the indication for eczema was selected by searching three drug databases and confirmed by contacting the manufacturers. RCTs for the treatment of eczema with CPM were selected in four Chinese literature databases and four English literature databases from their inception to August 31, 2019. The reporting quality of included RCTs was assessed based on the CONSORT-CHM formulas 2017. A univariate analysis was conducted to identify the factors associated with the reporting quality.

**Results:**

A total of 70 circulating CPMs had the indication for eczema. Among them, 21 CPMs with 144 RCTs reached the eligible criteria. The mean overall quality score (OQS) of 144 RCTs was 19.85 ± 2.73, which was much less than the maximum score of 38. Of the 38 items, 12 items were reported in over 70% of the trials, 6 items were reported in 50%–70% of the trials, and 16 items were reported in less than 50% of the trials. Publication after 2015 (*P* < 0.001) and the first author from a university hospital (*P*=0.010) were associated with the better reporting quality.

**Conclusion:**

There are a lot of circulating CPMs with the indication for eczema in mainland China, but both the quantity and the reporting quality of RCTs regarding those CPMs are suboptimal. It is necessary that authors and journal editors learn and adhere to the CONSORT-CHM formulas 2017 to enhance the reporting quality of RCTs for the treatment of eczema with CPM.

## 1. Introduction

Traditional Chinese medicine (TCM), including Chinese herbal medicine (CHM), acupuncture, cupping, and massage, plays an important role in the Chinese health care system, and almost all hospitals in China provide TCM services. Patients around the world also benefit from TCM extract, such as artemisinin, an antimalarial drug [1]. As an important part of CHM, Chinese patent medicine (CPM) is a production of a fixed formula of TCM processed in accordance with the prescribed prescriptions and standards and approved by China National Medical Products Administration (NMPA) [2]. Eczema is a common chronic, inflammatory skin disease, which presents as redness, scaling, swelling, and skin thickening. There is wide variation in the prevalence of eczema over the world. It is reported that 9.5% of children and 10.2% of adults are affected by eczema in the United States [3, 4]. In China, the prevalence of childhood eczema ranges from 11.8% to 18.71% [5, 6]. Compared with western medicine, such as topical corticosteroids and oral corticosteroids, CPM preparations are compound and could treat eczema through multiple mechanisms. In addition, there is no need to worry about adverse effects of glucocorticoids. Owing to these advantages, CPM has been widely used for eczema in mainland China for decades. Some studies have showed that CPM has good effects on alleviating pruritus and reducing skin lesions [7, 8]. However, there is no current investigation evaluating the reporting quality of these studies.

The randomized controlled trials (RCTs), providing the evidence of efficacy and safety of interventions, are fundamental to generate evidence-based practice guidelines. However, a reader cannot accurately evaluate the conclusion if a clinical trial is not reported with sufficient details. To standardize and improve the reporting quality of RCTs, the CONSORT (Consolidated Standards of Reporting Trials) statement was published in 1996 and updated in 2010 by the CONSORT group [9, 10]. It provides authors with a minimum set of recommendations for reports. The CONSORT statement has substantially improved the reporting quality of RCTs, including dermatology RCTs [11, 12]. The CONSORT-CHM formulas 2017 (Consolidated Standards of Reporting Trials for Chinese herbal medicine formulas 2017), a special extension for the CONSORT statement, has been developed in 2017 to guide the reporting of RCTs using CHM formulas [13].

To the best of our knowledge, the number of CPMs for eczema and the reporting quality of RCTs for the treatment of eczema with CPM in mainland China have not been investigated yet. The aims of this study are to search the circulating CPMs with the indication for eczema in mainland China and to evaluate the reporting quality of RCTs regarding these products by using the CONSORT-CHM formulas 2017.

## 2. Methods

This study consisted of three parts. First, circulating CPM with the indication for eczema in mainland China was selected by searching drug databases and confirmed by contacting the manufacturers. Second, RCTs of each eligible CPM were selected by retrieving literature databases. Third, the reporting quality of each included RCT was assessed based on the CONSORT-CHM formulas 2017.

### 2.1. Databases, Search Strategy, and Eligibility of CPM

By using the search terms “eczema” and “dermatitis,” CPM was collected from the following three drug databases: Sanjiu Yaopintong (https://ypk.39.net), Yaozhi database (https://db.yaozh.com/), and China NMPA (https://www.nmpa.gov.cn). CPM was included if it had the indication for eczema and drug approval number from China NMPA. CPM containing western medicine or out of circulation on the market was excluded.

### 2.2. Databases, Search Strategy, and Eligibility of RCTs

Four Chinese literature databases and four English literature databases were used to search relevant articles from their inception to August 31, 2019, including Chinese National Knowledge Infrastructure database (CNKI), Wanfang Data knowledge service platform (Wanfang), Chongqing Weipu database (VIP), Chinese Biomedical Literature database (SinoMed), PubMed, Cochrane Library, Embase, and Web of Science. The search strategy used for each database was ((“eczema” [Mesh] OR “eczema” [Title/Abstract]) OR (“dermatitis” [Mesh] OR “dermatitis” [Title/Abstract])) AND (“the name of each CPM” [Title/Abstract]). Inclusion criteria were as follows: RCTs published in Chinese or English; participants diagnosed as eczema, regardless of gender, age, or duration of eczema; the experimental group treated with CPM alone or CPM combined with western medicine same as the control group; and the control group treated with placebo or western medicine. Reviews, animal experiments, case reports, non-RCTs, duplicate studies, unavailable full texts, and studies with inappropriate interventions were excluded.

### 2.3. Data Extraction and Assessment of Reporting Quality

The following information from included RCTs was extracted: year of publication, language of the study, number of research center, sample size, and the type of the first author's hospital. The reporting quality was assessed by using the CONSORT-CHM formulas 2017, comprising a checklist of 38 items [13]. The full version of the CONSORT-CHM formulas 2017 is provided in Supplementary Table 1. Each item was given a “Yes, No, or Not applicable” response depending on the level of reporting of each RCT. In addition, to calculate the quality score of each RCT, “Yes” or “Not applicable” was scored 1 and “No” was scored 0. The overall quality score (OQS) of each RCT was calculated by adding together the score of each item and could vary between 0 and 38.

All procedures of CPM search, RCT search, data extraction, and assessment of reporting quality were carried out by two authors (ML and BYZ) independently, and discrepancies were resolved by consultation from the third author (LHZ).

### 2.4. Statistical Analysis

The primary outcomes were the number of circulating CPMs with the indication for eczema in mainland China and the mean OQS of included RCTs in accordance with the CONSORT-CHM formulas 2017. The secondary outcomes were the reporting rate of each item of the CONSORT-CHM formulas 2017 and the associations between the reporting quality and the following factors: year of publication and the type of the first author's hospital.

Continuous variables were presented as mean ± standard deviation (SD), and the independent sample *t*-test was used for comparison of continuous variables. Categorical variables were presented as rates, and comparison of categorical variables was performed by the chi-squared test. Mean differences (MDs) and their 95% confidence intervals (CIs) were calculated to illustrate differences in the OQS. Odds ratios (ORs) and their 95% CIs were calculated to illustrate differences in the reporting rate of each item. SPSS 22.0 software (SPSS Inc., Chicago, IL, USA) was used for data analysis, and *P* < 0.05 was considered statistically significant.

## 3. Results

### 3.1. Search Results of CPM for Eczema and Related RCTs

After removing the duplicates, 136 CPMs were selected from three drug databases. Of those, 46 CPMs without the indication for eczema, 4 CPMs without drug approval number from China NMPA, and 16 CPMs containing western medicine were excluded. Finally, 70 CPMs, including 37 topical CPMs and 33 oral CPMs, met the eligible criteria. The list of 70 included CPMs is summarized in Supplementary Table 2.

**Figure 1:**
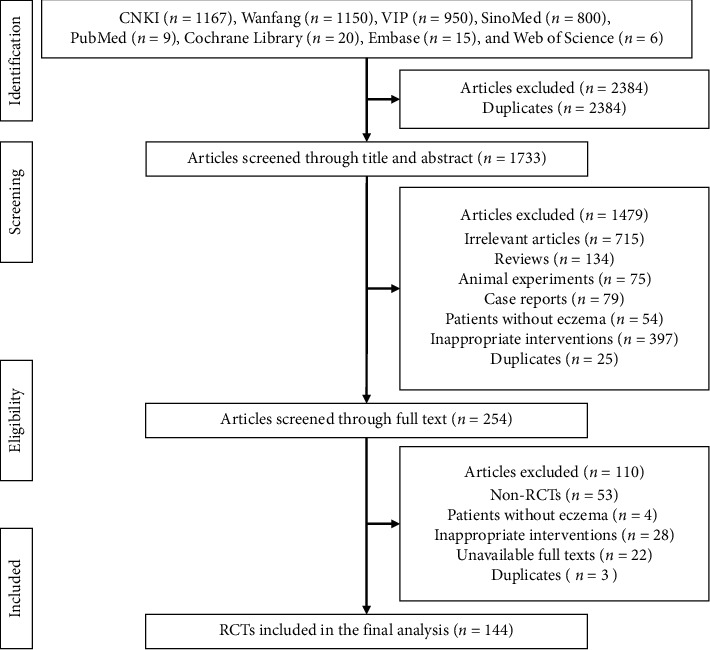
The flowchart of RCTs selection.

In total, 1733 articles were retrieved from four Chinese literature databases and four English literature databases. 1479 articles were excluded as a result of checking titles and abstracts. After screening full texts, 110 articles were excluded. 144 eligible RCTs which involved 21 CPMs were included in the final analysis. 11 out of 21 CPMs (52.38%) had no more than two eligible RCTs.  Figure 1 shows the flowchart of RCTs selection.  Table 1 provides the list of 21 included CPMs and the number of eligible RCTs of each CPM. The list of 144 included RCTs is shown in Supplementary File 1.

### 3.2. Characteristics of Included RCTs

All included RCTs were conducted by authors from mainland China. The 144 studies were published from 1996 to 2019, and 50 (34.72%) of them were published in recent five years. Only one article (0.69%) was printed in English in an international journal, and the remaining 143 articles were printed in Chinese in Chinese journals. Except for four multiple-center RCTs, most (97.22%, *n* = 140) were conducted at a single center. The mean sample size of all studies was 102.67 ± 49.25, ranging from 42 to 426. The first author was from a university hospital in 73 studies (50.69%).  Table 2 provides the general characteristics of 144 included RCTs. The detailed information about each included RCT, such as first author, sample size, interventions, and outcomes, is summarized in Supplementary Table 3.

### 3.3. Reporting Quality of Included RCTs

In the 144 RCTs, the mean OQS was 19.85 ± 2.73, ranging from 13 to 32. Three studies (2.08%) scored 27 or more, the OQS of 106 studies (73.61%) varied between 19 and 26, and 35 studies (24.31%) scored 18 or less. The distribution of OQS of 144 RCTs is shown in Table 3.

In the subgroup of 38 items, 12 items (3a, 4b, 5, 6a, 12a, 13a, 13b, 14a, 16, 18, 19, and 22) were reported in over 70% of the RCTs, and 6 items (1b, 2a, 4a, 11b, 12b, and 21) were stated in 50%–70% of the RCTs. Reporting rates were less than 50% for 16 items (1a, 1c, 2b, 7a, 8a, 8b, 9, 10, 11a, 15, 17a, 17b, 20, 23, 24, and 25). Among them, 2 items (17a and 17b) were not mentioned at all. In addition, 4 items (3b, 6b, 7b, and 14b) were not applicable in all studies. In the 144 studies, six studies (4.17%) adopted the blinding methods, including four double-blinded studies, one evaluator-blinded study, and one single-blinded study with an uncertain blinded object. Six studies (4.17%) adopted subgroup analysis to evaluate the effect of CPM for different stages of eczema or participants with different ages.  Table 4 presents the reporting number and percentage for each item of the CONSORT-CHM formulas 2017. The score of each item for each RCT is provided in Supplementary Table 4.

### 3.4. Reporting Quality by Year of Publication

The mean OQS of studies published after 2015 was significantly higher than that of studies published before 2015 (21.28 ± 2.76 vs 19.10 ± 2.41, MD: 2.18, 95% CI: 1.31–3.06, *P* < 0.001). According to the calculated ORs, the reporting rates of seven items were significantly higher in studies published after 2015: abstract (OR: 4.98, 95% CI: 1.93–12.83), background (OR: 8.90, 95% CI: 2.96–26.73), objectives (OR: 2.99, 95% CI: 1.36–6.55), methods used to generate the random allocation sequence (OR: 2.33, 95% CI: 1.06–5.12), statistical methods used to compare groups (OR: 4.17, 95% CI: 1.36–12.76), dates defining the periods of recruitment and follow-up (OR: 3.31, 95% CI: 1.07–10.25), and limitations (OR: 6.27, 95% CI: 1.22–32.34).  Table 5 presents the reporting quality of each item by year of publication.

### 3.5. Reporting Quality by the Type of the First Author's Hospital

The mean OQS of studies with the first author from a university hospital was significantly higher than that of studies with the first author from a nonuniversity hospital (20.42 ± 3.16 vs 19.27 ± 2.08, MD: 1.16, 95% CI: 0.28–2.04, *P*=0.010). The reporting rates of four items were significantly higher in the studies with the first author from a university hospital: objectives (OR: 2.67, 95% CI: 1.19–5.99), dates defining the periods of recruitment and follow-up (OR: 4.08, 95% CI: 1.52–10.95), baseline data, and funding. However, the reporting rate of abstract was lower in studies with the first author from a university hospital (OR: 0.47, 95% CI: 0.23–0.97).  Table 5 presents the reporting quality of each item by the type of the first author's hospital.

## 4. Discussion

In this study, we first summarized the number of circulating CPM with the indication for eczema in mainland China. Because most RCTs in mainland China did not have “TCM” or “CPM” in title, abstract, or keyword, we decided to search eligible CPM before retrieving literatures, rather than searching literature directly by using search terms “TCM” and “CPM.” As the result showed, 70 circulating CPMs had the indication for eczema in mainland China, and more than a third of eligible RCTs were published in recent five years, which demonstrated the wide application of CPM for eczema in mainland China. However, only 30% of included CPMs had the eligible RCTs, and 10 out of 21 CPMs had more than two eligible RCTs. The low proportions indicated that more efforts were needed to enhance the clinical evidence of CPM for eczema.

We also investigated the reporting quality of RCTs for the treatment of eczema with CPM by using the CONSORT-CHM formulas 2017, which added one item of “keywords” on the basis of the CONSORT statement. Our results showed that the mean OQS of 144 included RCTs was 19.85, only 52.24% of the maximum score (19.85/38), and few trials scored more than 70% of the maximum score. Similarly, a study on RCTs of TCM for diabetes mellitus in three top TCM journals showed that the total reporting rate of the CONSORT statement was only 45.0% [14]. On the other hand, only 12 items had an optimal reporting rate (reported in over 70% of trials) in our study. Some studies on RCTs of TCM treatments hold the same results, and the number of items with an optimal reporting rate ranged from 10 to 16 [15–17]. In summary, most included RCTs in this study did not strictly comply with the CONSORT-CHM formulas 2017, and the reporting quality of 144 RCTs was suboptimal. Low reporting quality could not only affect other researchers to understand the conclusion of a study but also increase the risk of bias of a systematic review and lower the level of clinical evidence. Therefore, authors and journal editors need to learn the items of the CONSORT-CHM formulas 2017 and apply it to article structures to improve the reporting quality.

The result showed that there were 16 items with a poor report rate (reported in below 50% of trials). Among them, some items are important and essential. When a trial has “randomized” or “RCT” in the title and keyword, it is easily retrieved and identified as a RCT. However, the proportions of two items were 2.78% and 3.47%, respectively, in this study. Other similar studies showed that 0.80%–5.9% of RCTs of TCM treatments mentioned randomization in the title [16, 18]. However, after applying the CONSORT statement, this proportion in a TCM journal was up to 100% [19].

Methodological items are pivotal elements for clinical trials. Sample size estimation can decrease the risk of false-negative results and obtain precise outcomes. The previous study showed that 50–200 subjects would need to be enrolled to reduce the risk of missing a true difference in cutaneous surgery therapeutic trials [20]. Although most included studies contained sixty or more patients, only one article (0.69%) described how sample size was determined. Similarly, this proportion was only 0.42% in 2861 RCTs of TCM treatments in CNKI database [18]. Instead, 52 out of 181 RCTs (28.73%) in the 44 international dermatology journals calculated the sample size [21]. In clinical trials, abundant participants may be a waste of time and money, while better results may not be achieved. Therefore, it needs a balance between statistics and expenses when considering the sample size of a trial.

Randomization is the major hallmark of a RCT. Its ultimate objectives are to create balanced treatment groups and to minimize the risk of biased results [22]. Inadequate randomization methods may exaggerate the estimate of the intervention effect [23]. In our study, 23.61% of the trials described sequence generation methods and 2.78% of the trials reported the types of randomization. Moreover, only one trial (0.69%) depicted allocation concealment and implementation. In many top TCM journals, the reporting rates of sequence generation methods were up to 47%–100%, whereas few RCTs reported allocation concealment and implementation [14, 17, 19]. Instead, international journals performed better in this respect. A review on 109 RCTs of eczema treatments in the Global Resource of Eczema Trials (GREAT) database from 2007 to 2011 found that 44 trials (40.37%) and 15 trials (13.76%) reported the randomization method and allocation concealment, respectively [24]. Among 141 RCTs in four top dermatology journals from 2015 to 2017, 70.21%, 58.16%, and 48.94% of the trials provided information on the methods of random sequence generation, allocation concealment, and implementation, respectively [12].

Blinding is considered as an essential component for internal validity of RCTs. It aims to prevent bias associated with expectations from patients, investigators, and assessors. Treatment effects may be overestimated or underestimated without blinding [25, 26]. Five trials (3.47%) in this study reported the blinding methods, and three of six trials (50%) described the similarities of interventions. Similarly, about 5% of RCTs of TCM treatments published in Chinese reported the blinding methods [14, 17], while this proportion increased to 30% in a TCM journal after the journal adopted the CONSORT statement [19]. In contrast, among 109 RCTs of eczema treatments in the GREAT database from 2007 to 2011, blinding was reported in 31.19% of the trials [24]. This percentage rose to 81.56% in 141 RCTs in four dermatology journals from 2015 to 2017, and 61.76% of the trials described the similarities of interventions [12].

Discrepancies between registered and published outcomes in clinical trials are common, which may make results attractive but also fake [27, 28]. Trial registrations and available protocols could provide some feasible solutions [29, 30]. There were one registered RCT (0.69%) and two RCTs (1.39%) with available protocols in this study. Almost RCTs of TCM treatments in TCM journals, regardless in Chinese or English, also lacked the information on these two items [18, 19]. On the contrary, 16.51% of 109 RCTs of eczema treatments in the GREAT database from 2007 to 2011 submitted the registration before the trial end date [24], and this proportion increased to 73.76% in 141 RCTs in four top dermatology journals from 2015 to 2017 [12]. Besides, funding source is an important part of a trial. It is more likely to report favorable outcomes for industry-sponsored studies [31]. In our study, only 6.25% of the trials reported the funding source, whereas nearly 70% of dermatology RCTs from PubMed database described the funding source [32].

In this study, some items had an optimal reporting rate, such as harms. The safety of a drug is as important as its effectiveness, and only safe treatments could be widely applied in clinical practice. In our study, 83.33% of the trials reported adverse events of CPM during the treatment. All adverse events were mild and tolerable, and no serious adverse events were observed. Analogously, harms were reported in 80 out of 110 RCTs (72.72%) in five top dermatology journals [33].

Like some previous studies [16, 18], our results showed that publication after 2015 was associated with the better reporting quality, representing as higher OQS and higher reporting rates of some important items, such as sequence generation methods and limitations. These findings demonstrated the efforts of authors in mainland China to improve the reporting quality. Another factor associated with the better reporting quality is the first author from a university hospital. In China, a university hospital, almost the top local hospital, has more advantages than a nonuniversity hospital in doctors' quality, medical equipment, and funding. In this study, the higher reporting rate of funding was found in the studies with the first author from a university hospital. However, the lower reporting rate of abstract in them may be due to short articles. In our study, short articles with one page were common, and abstract was simplified or omitted.

There are some limitations in this study. First, only CPMs with the indication for eczema were included. In clinical practice, numerous CPMs without the indication for eczema are used to treat eczema, such as Runzao Zhiyang capsule [8]. It is better to have an additional summary of these off-label CPMs. Second, only patients treated with placebo and western medicine were considered to be the control groups. Because of the lack of high-quality clinical evidence, TCM is not recommended for eczema in many guidelines outside China; therefore, we exclude the RCTs which made comparison between CPM and TCM.

## 5. Conclusions

In conclusion, there are a large number of CPMs with the indication for eczema in mainland China; however, both the quantity and the reporting quality of RCTs regarding those CPMs are suboptimal. Therefore, more well-designed clinical trials are needed to enhance the level of evidence of CPM for eczema. Authors and journal editors are encouraged to learn and adhere to the CONSORT-CHM formulas 2017 to improve the reporting quality of the RCTs.

## Figures and Tables

**Table 1:** The list of 21 included CPMs and the number of included RCTs of each CPM.

Category of CPM	No.	Name of CPM	Number of included RCTs
Topical CPM	1	Qingpeng ointment	45
	2	Chushi Zhiyang ointment	20
	3	Binghuang Fule ointment	19
	4	Wudai ointment	8
	5	Paeonol ointment	7
	6	Pifukang lotion	7
	7	Sophora alopecuroide oil liniment	6
	8	Erfukang liniment	6
	9	Qiangyue cream	6
	10	Meilu Xiaocuo ointment	2
	11	Geranium ointment	1
	12	Mayinglong musk hemorrhoid cream	1
	13	Chushi Zhiyang lotion	1
	14	Shuangzishen lotion	1
	15	Jieeryin lotion	1

Oral CPM	1	Xiaofeng Zhiyang granule	5
	2	Piminxiao capsule	2
	3	Phellodendron bark capsule	2
	4	Baixuanxiatare tablet	2
	5	Fangfeng Tongshen granule	1
	6	Sophora flavescens capsule	1

CPM, Chinese patent medicine; RCT, randomized controlled trial.

**Table 2:** General characteristics of 144 included RCTs.

Characteristics	Number (%) of RCTs
Year of publication	
2019–2015	50 (34.72%)
2014–2010	64 (44.45%)
2009–2005	25 (17.36%)
2004–1996	5 (3.47%)
Language	
Chinese	143 (99.31%)
English	1(0.69%)
Research centers	
Single center	140 (97.22%)
Multiple center	4 (2.78%)
Sample size	
<60	10 (6.94%)
60–100	82 (56.95%)
>100	52 (36.11%)
Type of the first author's hospital	
University hospital	73 (50.69%)
Nonuniversity hospital	71 (49.31%)

RCT, randomized controlled trial.

**Table 3:** The distribution of OQS of 144 included RCTs.

The distribution of OQS (percentage of maximum score of 38)	Number (%) of RCTs
38 (100%)	0 (0%)
37–35 (99%–90%)	0 (0%)
34–31 (89%–80%)	1 (0.69%)
30–27 (79%–70%)	2 (1.39%)
26–23 (69%–60%)	11 (7.64%)
22–19 (59%–50%)	95 (65.97%)
18–16 (49%–40%)	27 (18.75%)
15–12 (39%–30%)	8 (5.56%)

OQS, overall quality score; RCT, randomized controlled trial.

**Table 4:** The reporting number and percentage for each item of the CONSORT-CHM formulas 2017.

Section	Topic	Item number	*n*/*N*	%
Title, abstract, and keywords	Title	1a	4/144	2.78
	Abstract	1b	100/144	69.44
	Keywords	1c	5/144	3.47
Introduction	Background	2a	99/144	68.75
	Objectives	2b	35/144	24.31
Methods	Trial design	3a	142/144	98.61
		3b	0/0	0
	Participants	4a	82/144	56.94
		4b	123/144	85.42
	Interventions	5	114/144	79.17
	Outcomes	6a	106/144	73.61
		6b	0/0	0
	Sample size	7a	1/144	0.69
		7b	0/0	0
	Sequence generation	8a	34/144	23.61
		8b	4/144	2.78
	Allocation concealment mechanism	9	1/144	0.69
	Implementation	10	1/144	0.69
	Blinding	11a	5/144	3.47
		11b	3/6	50
	Statistical methods	12a	115/144	79.86
		12b	4/6	66.67
Results	Participant flow	13a	144/144	100
		13b	137/144	95.14
	Recruitment	14a	119/144	82.64
		14b	0/0	0
	Baseline data	15	8/144	5.56
	Numbers analyzed	16	144/144	100
	Outcomes and estimation	17a	0/144	0
		17b	0/143	0
	Ancillary analyses	18	6/6	100
	Harms	19	120/144	83.33
Discussion	Limitations	20	8/144	5.56
	Generalizability	21	74/144	51.39
	Interpretation	22	118/144	81.94
Other information	Registration	23	1/144	0.69
	Protocol	24	2/144	1.39
	Funding	25	9/144	6.25

CONSORT-CHM formulas, Consolidated Standards of Reporting Trials for Chinese herbal medicine formulas.

**Table 5:** Univariable analysis of reporting rate of each item of the CONSORT-CHM formulas 2017.

Item number	Year of publication		OR (95% CI)	*P* value	Type of the first author's hospital		OR (95% CI)	*P* value
	≥2015	<2015			University hospital	Nonuniversity hospital		
1a	2/50 (4%)	2/94 (2.13%)	1.92 (0.26–14.03)	0.906	3/73 (4.11%)	1/71 (1.41%)	3.00 (0.31–29.55)	0.632
1b	44/50 (88%)	56/94 (59.57%)	4.98 (1.93–12.83)	<0.001	45/73 (61.64%)	55/71 (77.46%)	0.47 (0.23–0.97)	0.039
1c	4/50 (8%)	1/94 (1.06%)	8.09 (0.88–74.43)	0.092	4/73 (5.48%)	1/71 (1.41%)	4.06 (0.44–37.23)	0.379
2a	46/50 (92%)	53/94 (56.38%)	8.90 (2.96–26.73)	<0.001	53/73 (72.60%)	46/71 (64.79%)	1.44 (0.71–2.92)	0.312
2b	19/50 (38%)	16/94 (17.02%)	2.99 (1.36–6.55)	0.005	24/73 (32.88%)	11/71(15.49%)	2.67 (1.19–5.99)	0.015
3a	50/50 (100%)	92/94 (97.87%)	NE	0.544	71/73 (97.26%)	71/71 (100%)	NE	0.497
3b	0/0 (0%)	0/0 (0%)	NE	NE	0/0 (0%)	0/0 (0%)	NE	NE
4a	32/50 (64%)	50/94 (53.19%)	1.56 (0.77–3.17)	0.212	44/73 (60.27%)	38/71 (53.52%)	1.32 (0.68–2.55)	0.413
4b	46/50 (92%)	77/94 (81.91%)	2.54 (0.81–8.01)	0.103	65/73 (89.04%)	58/71 (81.69%)	1.82 (0.71–4.71)	0.211
5	39/50 (78%)	75/94 (79.79%)	0.90 (0.39–2.08)	0.801	62/73 (84.93%)	52/71 (73.24%)	2.06 (0.90–4.72)	0.084
6a	33/50 (66%)	73/94(77.66%)	0.56 (0.26–1.19)	0.131	58/73 (79.45%)	48/71 (67.61%)	1.85 (0.87–3.94)	0.107
6b	0/0 (0%)	0/0 (0%)	NE	NE	0/0 (0%)	0/0 (0%)	NE	NE
7a	1/50 (2%)	0/94 (0%)	NE	0.347	1/73 (1.37%)	0/71 (0%)	NE	1.000
7b	0/0 (0%)	0/0 (0%)	NE	NE	0/0 (0%)	0/0 (0%)	NE	NE
8a	17/50 (34%)	17/94 (18.09%)	2.33 (1.06–5.12)	0.032	21/73 (28.77%)	13/71 (18.31%)	1.80 (0.82–3.96)	0.140
8b	2/50 (4%)	2/94 (2.13%)	1.92 (0.26–14.03)	0.906	4/73 (5.48%)	0/71 (0%)	NE	0.135
9	1/50 (2%)	0/94 (0%)	NE	0.347	1/73 (1.37%)	0/71 (0%)	NE	1.000
10	1/50 (2%)	0/94(0%)	NE	0.347	1/73 (1.37%)	0/71(0%)	NE	1.000
11a	1/50 (2%)	4/94 (4.26%)	0.46 (0.05–4.22)	0.821	5/73 (6.85%)	0/71 (0%)	NE	0.074
11b	1/1 (100%)	2/5 (40%)	NE	1.000	3/6 (50%)	0/0 (0%)	NE	NE
12a	46/50 (92%)	69/94 (73.40%)	4.17 (1.36–12.76)	0.008	60/73 (82.19%)	55/71 (77.46%)	1.34 (0.59–3.04)	0.479
12b	2/2 (100%)	2/4 (50%)	NE	0.467	2/3 (66.67%)	2/3(66.67%)	1.00 (0.03–29.81)	1.000
13a	50/50 (100%)	94/94 (100%)	NE	NE	73/73 (100%)	71/71 (100%)	NE	NE
13b	48/50 (96%)	89/94 (94.68%)	1.35 (0.25–7.21)	1.000	70/73 (95.89%)	67/71 (94.37%)	1.39 (0.30–6.46)	0.970
14a	46/50 (92%)	73/94 (77.66%)	3.31 (1.07–10.25)	0.031	67/73 (91.78%)	52/71 (73.24%)	4.08 (1.52–10.95)	0.003
14b	0/0 (0%)	0/0 (0%)	NE	NE	0/0 (0%)	0/0 (0%)	NE	NE
15	3/50 (6%)	5/94 (5.32%)	1.14 (0.26–4.96)	1.000	8/73 (10.96%)	0/71 (0%)	NE	0.012
16	50/50 (100%)	94/94 (100%)	NE	NE	73/73 (100%)	71/71 (100%)	NE	NE
17a	0/50 (0%)	0/94 (0%)	NE	NE	0/73 (0%)	0/71 (0%)	NE	NE
17b	0/50 (0%)	0/93 (0%)	NE	NE	0/72 (0%)	0/71 (0%)	NE	NE
18	2/2 (100%)	4/4 (100%)	NE	NE	3/3 (100%)	3/3 (100%)	NE	NE
19	44/50 (88%)	76/94 (80.85%)	1.74 (0.64–4.70)	0.273	60/73 (82.19%)	60/71 (84.51%)	0.85 (0.35–2.04)	0.709
20	6/50 (12%)	2/94 (2.13%)	6.27 (1.22–32.34)	0.038	5/73 (6.85%)	3/71 (4.23%)	1.67 (0.38–7.25)	0.746
21	29/50 (58%)	45/94 (47.87%)	1.50 (0.75–3.01)	0.247	35/73 (47.95%)	39/71 (54.93%)	0.76 (0.39–1.46)	0.402
22	45/50 (90%)	73/94 (77.66%)	2.59 (0.91–7.35)	0.067	58/73 (79.45%)	60/71 (84.51%)	0.71 (0.30–1.67)	0.430
23	1/50 (2%)	0/94 (0%)	NE	0.347	1/73 (1.37%)	0/71 (0%)	NE	1.000
24	2/50 (4%)	0/94 (0%)	NE	0.119	2/73 (2.74%)	0/71 (0%)	NE	0.497
25	6/50 (12%)	3/94 (3.19%)	4.14 (0.99–17.32)	0.086	9/73 (12.33%)	0/71 (0%)	NE	0.007

CONSORT-CHM formulas, Consolidated Standards of Reporting Trials for Chinese herbal medicine formulas; CI, confidence interval; OR, odds ratio; NE, not estimable.

## Data Availability

The data used to support the results of this study are included within the article and supplementary materials.

## Supplementary Materials

Supplementary Materials Supplementary Table 1: checklist of items for the CONSORT-CHM formulas 2017. Supplementary Table 2: the list of Chinese patent medicine with the indication for eczema. Supplementary File 1: the list of 144 randomized controlled trials on Chinese patent medicine for eczema. Supplementary Table 3: the characteristics of 144 randomized controlled trials on Chinese patent medicine for eczema. Supplementary Table 4: the score of each item for 144 randomized controlled trials based on the CONSORT-CHM formulas 2017.

## References

[1] Tu Y. (2016). Artemisinin-a gift from traditional Chinese medicine to the world (nobel lecture). *Angewandte Chemie International Edition*.

[2] Sun S., Mo X. W., Li Y. F. (2019). Effectiveness comparisons of Chinese patent medicine on treating premature ejaculation: a systematic review and meta-analysis. *Medicine (Baltimore)*.

[3] Drury K. E., Schaeffer M., Silverberg J. I. (2016). Association between atopic disease and anemia in US children. *JAMA Pediatrics*.

[4] Jonathan J. I., Hanifin J. M. (2013). Adult eczema prevalence and associations with asthma and other health and demographic factors: a US population-based study. *The Journal of Allergy and Clinical Immunology*.

[5] Song N., Shamssain M., Zhang J. (2014). Prevalence, severity and risk factors of asthma, rhinitis and eczema in a large group of Chinese schoolchildren. *Journal of Asthma*.

[6] Guo Y. F., Li P., Tang J. P. (2017). Prevalence of skin diseases in pre-school children aged 0-7 years in 12 cities of China. *Chinese Journal of Dermatology*.

[7] Li Y., Xu W., Li L. F., Zhang R. N. (2019). Antipruritic effect of qingpeng ointment on the localized nonexudative eczema. *Evidence-Based Complementary and Alternative Medicine*.

[8] Huang D., Chen K., Zhang F.-R. (2019). Efficacy and safety of Run Zao Zhi Yang capsule on chronic eczema: a multiple-center, randomized, double-blind, placebo-controlled clinical study. *Journal of Dermatological Treatment*.

[9] Begg C., Cho M., Eastwood S. (1996). Improving the quality of reporting of randomized controlled trials. The CONSORT statement. *JAMA: The Journal of the American Medical Association*.

[10] Schulz K. F., Altman D. G., Moher D., CONSORT Group (2010). CONSORT 2010 statement: updated guidelines for reporting parallel group randomised trials. *BMJ*.

[11] Hopewell S., Dutton S., Yu L. M., Chan A. W., Altman D. G. (2010). The quality of reports of randomised trials in 2000 and 2006: comparative study of articles indexed in PubMed. *BMJ*.

[12] Kim D. Y., Park H. S., Cho S., Yoon H. S. (2019). The quality of reporting randomized controlled trials in the dermatology literature in an era where the CONSORT statement is a standard. *British Journal of Dermatology*.

[13] Cheng C.-W., Wu T.-X., Shang H.-C. (2017). CONSORT Extension for Chinese herbal medicine formulas 2017: recommendations, explanation, and elaboration. *Annals of Internal Medicine*.

[14] Wang P., Xu Q., Sun Q., Fan F. F., Guo X. R., Guo F. (2013). Assessment of the reporting quality of randomized controlled trials on the treatment of diabetes mellitus with traditional Chinese medicine: a systematic review. *PLoS One*.

[15] Liu K., Zeng J., Pei W. (2019). Assessing the reporting quality in randomized controlled trials of acupuncture for postherpetic neuralgia using the CONSORT statement and STRICTA guidelines. *Journal of Pain Research*.

[16] Zhao X., Zhen Z., Guo J. (2016). Assessment of the reporting quality of placebo-controlled randomized trials on the treatment of type 2 diabetes with traditional Chinese medicine in mainland China: a PRISMA-compliant systematic review. *Medicine (Baltimore)*.

[17] Fan F. F., Xu Q., Sun Q., Zhao S. J., Wang P., Guo X. R. (2014). Assessment of the reporting quality of randomized controlled trials on treatment of coronary heart disease with traditional Chinese medicine from the Chinese journal of integrated traditional and Western medicine: a systematic review. *PLoS One*.

[18] Li J., Liu Z., Chen R. (2014). The quality of reports of randomized clinical trials on traditional Chinese medicine treatments: a systematic review of articles indexed in the China National Knowledge Infrastructure database from 2005 to 2012. *BMC Complementary and Alternative Medicine*.

[19] Liu X. T., Zhang X., Wen S., Peng L., Hong Q., Kang D. (2015). Impact of the Consolidated Standards of Reporting Trials (CONSORT) checklist on reporting of randomized clinical trials in traditional Chinese medicine. *Journal of Evidence-Based Medicine*.

[20] Alam M., Barzilai D. A., Wrone D. A. (2005). Power and sample size of therapeutic trials in procedural dermatology. *Dermatologic Surgery*.

[21] McClean M., Silverberg J. I. (2015). Statistical reporting in randomized controlled trials from the dermatology literature: a review of 44 dermatology journals. *British Journal of Dermatology*.

[22] Broglio K. (2018). Randomization in clinical trials. *JAMA*.

[23] Savović J., Jones H. E., Altman D. G. (2012). Influence of reported study design characteristics on intervention effect estimates from randomized, controlled trials. *Annals of Internal Medicine*.

[24] Nankervis H., Baibergenova A., Williams H. C., Thomas K. S. (2012). Prospective registration and outcome-reporting bias in randomized controlled trials of eczema treatments: a systematic review. *Journal of Investigative Dermatology*.

[25] Saltaji H., Armijo-Olivo S., Cummings G. G., Amin M., da Costa B. R., Flores-Mir C. (2018). Influence of blinding on treatment effect size estimate in randomized controlled trials of oral health interventions. *BMC Medical Research Methodology*.

[26] Armijo-Olivo S., Fuentes J., da Costa B. R., Saltaji H., Ha C., Cummings G. G. (2017). Blinding in physical therapy trials and its association with treatment effects. *American Journal of Physical Medicine & Rehabilitation*.

[27] Liu J. P., Han M., Li X. X. (2013). Prospective registration, bias risk and outcome-reporting bias in randomised clinical trials of traditional Chinese medicine: an empirical methodological study. *BMJ Open*.

[28] Howard B., Scott J. T., Blubaugh M., Roepke B., Scheckel C., Vassar M. (2017). Systematic review: outcome reporting bias is a problem in high impact factor neurology journals. *PLoS One*.

[29] Calméjane L., Dechartres A., Tran V. T., Ravaud P. (2018). Making protocols available with the article improved evaluation of selective outcome reporting. *Journal of Clinical Epidemiology*.

[30] Jones C. W., Keil L. G., Holland W. C., Caughey M. C., Platts-Mills T. F. (2015). Comparison of registered and published outcomes in randomized controlled trials: a systematic review. *BMC Medicine*.

[31] Riaz H., Raza S., Khan M. S., Riaz I. B., Krasuski R. A. (2015). Impact of funding source on clinical trial results including cardiovascular outcome trials. *The American Journal of Cardiology*.

[32] Charrow A., Xia F. D., Joyce C., Mostaghimi A. (2017). Diversity in dermatology clinical trials. *JAMA Dermatology*.

[33] Haddad C., Sigha O. B., Lebrun-Vignes B., Chosidow O., Fardet L. (2017). Reporting of harm and safety results in randomized controlled trials published in 5 dermatology journals. *Journal of the American Academy of Dermatology*.

