# Supplementary material for: Reporting Quality of Randomized Controlled Trials for the Treatment of Eczema with Chinese Patent Medicine Based on the CONSORT-CHM Formulas 2017
Source: Evid Based Complement Alternat Med. 2020 Sep 14;2020:2949125. doi: 10.1155/2020/2949125 (PMC7512083; doi:10.1155/2020/2949125)
Supplement: Supplementary Materials — Supplementary Table 1: checklist of items for the CONSORT-CHM formulas 2017. Supplementary Table 2: the list of Chinese patent medicine with the indication for eczema. Supplementary File 1: the list of 144 randomized controlled trials on Chinese patent medicine for eczema. Supplementary Table 3: the characteristics of 144 randomized controlled trials on Chinese patent medicine for eczema. Supplementary Table 4: the score of each item for 144 randomized controlled trials based on the CONSORT-CHM formulas 2017. [file 2949125.f1.zip › 2949125.f1/Supplementary File 1 The list of 144 randomized controlled trials (2).docx]

Supplementary File 1: The list of 144 randomized controlled trials on Chinese patent medicine for eczema.

[1] Y. Li, W. Xu, L.F. Li, and R.N. Zhang, “Antipruritic effect of Qingpeng ointment on the localized nonexudative eczema,” *Evidence-based complementary and Alternative Medicine*, 2019, Article ID 4961691, 2019.

[2] G. Wang and T.T. Li, “Clinical efficacy observation of Qingpeng ointment in the treatment of localized eczema in children,” *Chinese Journal of Clinical Rational Drug Use*, vol. 11, no. 20, pp. 83-84, 2018.

[3] J.B. Zhang and S.J. Chen, “Clinical observation of Qingpeng ointment in the treatment of children with localized eczema, *Chinese Journal of Clinical Rational Drug Use*, vol. 10, no. 14, pp. 80-81, 2017.

[4] Y.Y. She, S.J. Chen, C.H. Yao, Q.Y. Liu, Y. Qu, and J.J Wang, “Efficacy observation of Qingpeng ointment in the treatment of localized eczema in children,” *Journal of Clinical Medical Literature*, vol. 3, no. 44, pp. 8699-8700, 2016.

[5] C.Z. Dong and C.X. Qiu, “Clinical observation of Qingpeng ointment in the treatment of chronic eczema,” *Modern Women (Frontiers Medicine)*, no. 006, pp. 27-28, 2014.

[6] J. Zeng and Y.R. Yang, “Clinical observation of Qingpeng ointment in the treatment of chronic eczema,” *The Journal of Practical Medicine*, vol. 29, no. 17, pp. 2932-2933, 2013.

[7] H. Tang, Q.P. Yang, D. Luo et al., “Qingpeng ointment in the treatment of eczema: a multi-center, randomized, double-blind, placebo-controlled study,” *Chinese Journal of Dermatology*, vol. 44, no. 12, pp. 838-841, 2011.

[8] S.J. Chen, Y.L. Song, Q.Y. Liu et al., “Efficacy observation of Qingeng ointment in the treatment of chronic eczema,” *China Practical Medical*, vol. 05, no. 27, pp. 72-73, 2010.

[9] L. Gao and L.T. Zhao, “Clinical observation of ebastine combined with Qingpeng ointment in the treatment of eczema,” *Clinical Journal of Chinese Medicine*, vol. 7, no. 30, pp. 87-88, 2015.

[10] Y. Yang, S. Wang, X. Li, and L.D. Zhu, “Clinical efficacy of Qingpeng ointment combined with desonide ointment in the treatment of atopic dermatitis in children,” *Medical Information*, vol. 31, no. 11, pp. 130-132, 2018.

[11] Y.F. He, “Efficacy observation of Qingpeng ointment in the treatment of eczema in infants,” *The Medical Forum*, vol. 20, no. 34, pp. 4874-4875, 2016.

[12] Y.Q. Li, F. Yang, and X.B. Liu, “Recent and forward efficacy analysis on halogen betamethasone cream combined with Qingpeng ointment in treatment of localized eczema,” *Drug Evaluation Research*, vol. 39, no. 2, pp. 286-288, 2016.

[13] W.J. Hou, “Clinical observation of integrated traditional Chinese and western medicine in the treatment of 35 cases of scrotal eczema,” *Henan Traditional Chinese Medicine*, vol. 34, no. 12, pp. 2414-2415, 2014.

[14] Y.F. Zhang, X.F. Ping, and S.Y. Jia, “Clinical observation of Qingpeng ointment combined with hydrocortisone butyrate cream in the treatment of 43 cases of chronic eczema,” *The Chinese Journal of Dermatovenereology*, vol. 28, no. 8, pp.876-878,879, 2014.

[15] J.W. Zheng, L.X. Zhang, Y.P. Bai, and D.Q. Yang, “Clinical observation of sequential therapy of mometasone furoate cream with Qingpeng ointment in the treatment of 44 cases of chronic eczema,” *Guiding Journal of Traditional Chinese Medicine and Pharmacology*, vol. 18 no. 9, pp. 37-39, 2012.

[16] X.X. Zhai, “Efficacy observation of Qingpeng ointment combined with fluticasone propionate cream in the treatment of chronic eczema,” *Chinese Journal of Aesthetic Medicine*, vol. 20, no. z2, pp. 470, 2011.

[17] G.H. Peng, S.J. Zhang, J.S. Wang, and H.Y. Wang, “Clinical observation of Qingpeng ointment combined with triamcinolone acetonide and econazole cream in the treatment of palmoplantar keratotic eczema,” *Hebei Medical Journal*, vol. 30, no. 20, pp. 3178-3179, 2011.

[18] J.Q. Jiang, “Clinical efficacy observation of Qingpeng ointment combined with mometasone furoate cream in the treatment of chronic eczema,” in *Proceedings of the Chinese 2017 Annual Academic Conference on Leprosy and Skin Disease*, Xingyi, Guizhou, China, August 2017.

[19] X.H. Wang, “Efficacy of Qingpeng ointment combined with hydrocortisone butyrate cream in the treatment of eczema,” *Chinese Journal of Primary Medicine and Pharmacy*, vol. 22, no. 14, pp. 2159-2161, 2015.

[20] L. Zhou, “Efficacy observation of Qingpeng ointment combined with halometasone cream in the treatment of chronic eczema,” *Chinese Journal of Aesthetic Medicine*. vol. 20, no. z2, pp. 454-455, 2011.

[21] L.Q. Yu, “Clinical observation of Qingpeng ointment combined with desonide cream in the treatment of chronic eczema,” *China Higher Medical Education*, no. 7, pp. 143-144, 2018.

[22] Y.R. Liu and Y.T. Mao, “Efficacy observation of sequential therapy of halometasone cream and Qingpeng ointment in the treatment of chronic eczema,” *Yi Yao Qian Yan*, vol. 4, no. 21, pp. 385-386, 2014.

[23] J.Q. Li, D.H. Liang, and X. Xu, “Efficacy on sequential therapy of fluticasone propionate cream with Qingpeng ointment in the treatment of chronic eczema,” *Chinese Journal of Dermatovenerology of Integrated Traditional and Western Medicine*, vol. 11, no. 6, pp. 360-361, 2012.

[24] S. Li, “Observation of the effect of 0.1% tacrolimus ointment on chronic eczema,” *Capital Medicine*, vol. 26, no. 12, pp. 69, 2019.

[25] R.Z. Teng, “Efficacy observation of Qingpeng ointment combined with hydrocortisone butyrate cream in the treatment of chronic eczema,” *China Journal of Leprosy and Skin Diseases*, vol. 29, no. 7, pp. 483, 2013.

[26] C.S. Zhang, “The observation of the curative effect of 0.1% tacrolimus ointment on chronic eczema of scrotum,” *Chinese and Foreign Medical Research*, vol. 16, no. 14, pp. 130-132, 2018.

[27] Y. Li, W. Xu, B.Q. Yang et al., “Qingpeng ointment for the treatment of localized eczema in Chinese children: a multicenter, randomized controlled study,” *Chinese Journal of Dermatology*, vol. 50, no. 6, pp. 412-416, 2017.

[28] K. Tang and S. Liang, “Analysis of efficacy of Qingpeng ointment in the treatment of dermatitis and eczema,” *Health Guide*, no. 20, pp. 184, 2017.

[29] S.H. Song, Y.H. Zhang, P. Chen, P. Liu, and Y.B. Liu, “Efficacy observation of Qingpeng ointment in the treatment of chronic eczema in children,” *Chinese Journal of Clinical Rational Drug Use*, vol. 10, no. 8, pp. 77-78, 2017.

[30] L.B. Kong, “Efficacy and safety of Qingpeng ointment in the treatment of eczema,” *Journal of Clinical Medical Literature*, vol. 4, no. 14, pp. 2707, 2017.

[31] J.L. Zhang, Z.L. Tan, and G.L. Jin, “Clinical observation of Qingpeng ointment in the treatment of dermatitis and eczema,” *Journal of Clinical Medical Literature*, vol. 3, no.51, pp. 10238,10259, 2016.

[32] J. Han, “Clinical efficacy of Qingpeng ointment for dermatitis and eczema,” *China Health Care and Nutrition*, vol. 26, no. 33, pp. 391-392, 2016.

[33] L.X. Zhang and X. Kou, “Clinical efficacy of Qingpeng ointment in the treatment of hand eczema,” *China Health Care and Nutrition*, vol. 26, no. 30, pp. 281, 2016.

[34] H. Yang, “Clinical observation of Qingpeng ointment in the treatment of dermatitis and eczema,” *Medical Information*, vol. 29, no. 30, pp. 222, 2016.

[35] S.W. Tang, S.Q. Xie, X. Song, Q.L. Wang, and Y. Shen, “Clinical efficacy of package therapy with Qingpeng ointment for chronic eczema,” *World Clinical Drugs*, vol. 37, no. 1, pp. 38-41,66, 2016.

[36] J.Z. Guo, E.S. Huang, Z.Z. Ye, W.W. Li, and X.Y. Lu, “Clinical observation of Qingpeng ointment for the treatment of localized eczema in children,” *Chinese Journal of Dermatology*, vol. 48, no. 12, pp. 895-896, 2015.

[37] J. Guo, W. He, and J.S. Guan, “Efficacy observation of Qingpeng ointment combined with halometasone cream for perianal eczema,” *Yi Yao Qian Yan*, vol. 5, no. 19, pp. 130-132, 2015.

[38] Y. Shen, “Efficacy of Qingpeng ointment for the treatment of mild to moderate atopic dermatitis,” *Journal of Mathematical Medicine*, vol. 28, no. 2, pp. 259-260, 2015.

[39] M. Gao, Y.Z. Tang, and D. Wang, “Efficacy observation of Qingpeng ointment for eczema of external auditory canal,” *Journal of Clinical Dermatology*, vol. 44, no. 3, pp. 185-186, 2015.

[40] D.Y. Huang, H.L. Shi, and Y. Chen, “Efficacy observation of Qingpeng ointment for neurodermatitis,” *China Pharmacy*, vol. 25, no. 43, pp. 4108-4110, 2014.

[41] Z.G. Zhao, F. Xie, Y.X. Ding, H. Zhao, and H.J. Li, “Clinical efficacy of Qingpeng ointment in the treatment of chronic eczema,” *Chinese Journal of Dermatology*, vol. 44, no. 11, pp. 813-814, 2011.

[42] H. Wang, G.S. Li, J.W. Long, X.M. Pi, and Y.Y. Wang, “Clinical observation of Qingpeng ointment in the treatment of eczema and dermatitis,” *The Chinese Journal of Dermatovenereology*, vol. 25, no. 5, pp. 404-405,410, 2011.

[43] W.H. Zeng, “Qingpeng ointment for the treatment of chronic eczema: a randomized controlled trial,” *China Practical Medical*, vol. 6, no. 12, pp. 129-130, 2011.

[44] J.B. Li, “Qingpeng ointment in the treatment of 38 cases of stasis eczema,” *Journal of China Traditional Chinese Medicine Information*, vol. 3, no. 10, pp. 280, 2011.

[45] Z.X. Sun, Y.Q. Li, Y.W. Li, R. Yao, and L. Zhang, “The clinical observation on the effect of Qingpeng ointment on 68 patients with subacute eczema,” *Medical Information*, vol. 5, no. 11, pp. 3273-3274, 2010.

[46] L.R. Liu and K. Qiu, “The influence of Chushi Zhiyang ointment combined with levocetirizine tablet on the efficacy and quality of life of chronic perianal eczema,” *Journal of Clinical Medicine in Practice*, vol. 21, no. 13, pp. 182-184, 2017.

[47] N. Wang, “Analysis of outcomes and efficacy of tacrolimus ointment in the treatment of dermatitis and eczema,” *China Practical Medical*, vol. 14, no. 6, pp. 111-112, 2019.

[48] C.P. Shen, L.H. Wang, H. Wang et al., “Efficacy and safety of Chushi Zhiyang ointment for the treatment of mild atopic dermatitis in infants: a multicenter clinical study,” *Chinese Journal of Dermatology*, vol. 50, no. 4, pp. 279-282, 2017.

[49] Q. Ran and X.L. Huang, “Efficacy and safety of Chushi Zhiyang ointment in the treatment of infantile eczema,” *Yi Yao Qian Yan*, vol. 7, no. 4, pp. 64-65, 2017.

[50] Y.T. Tang and W. Deng, “Effect of tacrolimus ointment on cellular immunity of patients with acute dermatitis and eczema,” *Medical Recapitulate*, vol. 22, no. 18, pp. 3739-3742, 2016.

[51] A.X. Huang, “Clinical efficacy observation of Chushi Zhiyang ointment in the treatment of eczema in children,” *Chinese Manipulation and Rehabilitation Medicine*, vol. 6, no. 16, pp. 36-37, 2015.

[52] R. Ding, “Efficacy observation of Chushi Zhiyang ointment in the treatment of perianal eczema,” *China Journal of Leprosy and Skin Disease*, vol. 30, no. 2, 121, 2014.

[53] J.M. Tao, “Efficacy observation of Chushi Zhiyang ointment and epinastine hydrochloride in the treatment of eczema,” *Health Must Read Magazine*, no. 12, pp. 395, 2012.

[54] Y. Shen, “Clinical observation of Chushi Zhiyang ointment in the treatment of vulvar eczema,” *Seek Medical and Ask Medicine*, vol. 10, no. 10, pp. 221-222, 2012.

[55] X.L. Huang, “Clinical observation of Chushi Zhiyang ointment in the treatment of neurodermatitis,” *Guide of China Medicine,* vol. 10, no. 29, pp. 18-19, 2012.

[56] L. Geng, Y.F. Wang, R. Xu et al., “Clinical efficacy observation of Chushi Zhiyang ointment in the treatment of scrotal eczema,” *Chinese Journal of Dermatovenerology of Integrated Traditional and Western Medicine*, vol. 9, no. 6, pp. 369-370, 2010.

[57] J.B. Zhang, H. Chen, L.Q. Wang et al., “Efficacy observation of Chushi Zhiyang ointment in the treatment of neurodermatitis,” *Medical Information*, vol. 23, no. 8, pp. 149-150, 2010.

[58] Y. Liu, “Clinical observation of Chushi Zhiyang ointment combined with triamcinolone acetonide and econazole cream for vulvar eczema,” *Journal of Practical Traditional Chinese Medicine*, vol. 32, no. 10, pp. 989, 2016.

[59] J. Wei, “Chushi Zhiyang ointment combined with desonide cream in the treatment of facial dermatitis: a controlled study,” *Journal of China Prescription Drug*, vol. 13, no. 12, pp. 66-67, 2015.

[60] C.F. Shen, “Efficacy observation of 0.05% desonide cream combined with Chushi Zhiyang ointment in the treatment of eczema in infants,” *Journal of Dermatology and Venereology*, vol. 36, no. 5, pp. 286-287, 2014.

[61] Y.H. Zhang and Y.H. Wang, “Clinical observation of Chushi Zhiyang ointment combined with hydrocortisone butyrate cream for acute eczema,” *Heilongjiang Journal of Traditional Chinese Medicine*, vol. 39, no. 5, pp. 18, 2010.

[62] C.R. Deng, “Clinical observation of Chushi Zhiyang ointment in the treatment of infantile eczema,” *China Journal of Leprosy and Skin Diseases*, vol. 25, no. 7, pp. 555, 2009.

[63] X.H. Liu, Y. Zhang, and S. Hu, “Analysis of desonide cream combined with Chushi Zhiyang ointment in the treatment of eczema in children,” *Modern Diagnosis and Treatment*, vol. 30, no. 10, pp. 1636-1637, 2019.

[64] C.R. Li, D.F. Du, and J. Zhang, “Efficacy observation of Chushi Zhiyang ointment combined with hydrocortisone butyrate cream in the treatment of perianal eczema,” *Zhejiang Journal of Traditional Chinese Medicine*, vol. 54, no. 07, pp. 509, 2019.

[65] B Li, “*The clinical Study on eczema gel in the treatment of chronic eczema*,” M.S. thesis, Hunan University of Chinese medicine, Changsha, China, 2015.

[66] X.Y. Zhu, “Clinical experience of Binghuang Fule ointment combined with loratadine tablet in the treatment of subacute and chronic hand eczema,” *For All health*, vol. 8, no. 6, pp. 523-524, 2014.

[67] X.Y. Liu, “Efficacy observation of Binghuang Fule ointment in the treatment of dermatitis and eczema,” *Chinese Journal of Ethnomedicine and Ethnopharmacy*, vol. 22, no. 9, pp. 95, 2013.

[68] S. Wu, L.H. Deng, Y.F. Hu, S.J. Liu, G. Zhao, and G.R. Chen, “Clinical observation of Binghuang Fule ointment combined with loratadine tablet in the treatment of subacute and chronic hand eczema,” *Journal of Jinan University (Natural Science and Medicine Edition)*, vol. 33, no. 6, pp. 608-611, 2012.

[69] F.J. Xu, “Effect of Binghuang Fule ointment combined with levocetirizine capsule in the treatment of chronic eczema,” *China Continuing Medical Education*, vol. 7, no. 32, pp. 195-196, 2015.

[70] Z.Y. Zhang, D. Niu, and B.R. Zhang, “Observation of the effect of Binghuang Fule ointment combined with loratadine tablet in the treatment of subacute and chronic hand eczema,” *Jin Ri Jian Kang*, vol. 13, no. 6, pp. 14, 2014.

[71] X. Liu, “Efficacy observation of two kinds of medicine in the treatment of chronic eczema,” *China Medical Cosmetology*, no. 5, pp. 121,125, 2014.

[72] L. Xu, X.Y. Tang, and Z.B. Yang, “Homemade eczema cream in the treatment of 40 cases of chronic eczema,” *Journal of External Therapy of Traditional Chinese Medicine*, vol. 21, no. 2, pp. 18-19, 2012.

[73] Y.Z. Chen, Y.G. Huang, and Q. Ou, “Clinical observation of Binghuang Fule ointment in the treatment of neurodermatitis,” *Modern Journal of Integrated Traditional Chinese and Western Medicine*, vol. 21, no. 9, pp. 941, 2012.

[74] C. Li, “Efficacy observation of Binghuang Fule ointment in the treatment of neurodermatitis,” *Chinese Medicine Modern Distance Education of China*, vol. 8, no. 24, pp. 30, 2010.

[75] H.J. Wang, J.A. Zhang, and J.B. Yu, “Clinical observation of Binghuang Fule ointment in the treatment of 48 cases of neurodermatitis,” *Chinese Journal of Dermatovenereology*, vol. 23, no. 3, pp. 191-192, 2009.

[76] X.L. Zhao, H. Wang, and S.J. Tao, “Efficacy observation of Binghuang Fule ointment in the treatment of neurodermatitis,” *China Journal of Leprosy and Skin Diseases*, vol. 24, no. 9, pp. 750-751, 2008.

[77] W.G. Wei and S.W. Zeng, “Efficacy observation of Binghuang Fule ointment for eczema,” *International Journal of Traditional Chinese Medicine*, vol. 29, no. 2, pp. 120, 2007.

[78] Z. Liu, “Efficacy observation of the treatment of chronic eczema,” *China Health Industry*, vol. 11, no. 12, pp. 174-175, 2014.

[79] Y.G. Yang, “Clinical observation of compound flumetasone ointment in the treatment of chronic eczema,” *Chinese Medicine Modern Distance Education of China*, vol. 10, no. 4, pp. 21-22, 2012.

[80] X.J. Yang, “Efficacy observation of Binghuang Fule ointment in the treatment of neurodermatitis and chronic eczema,” *China Journal of Leprosy and Skin Diseases*, vol. 22, no. 11, pp. 906, 2006.

[81] Z.B. Zhang and S. Liu, “Efficacy observation of Binghuang Fule ointment in the treatment of adult atopic dermatitis,” *Journal of Sichuan of Traditional Chinese Medicine*, vol. 26, no. 10, pp. 95, 2008.

[82] S.P. Liu, “Binghuang Fule ointment combined with Qumixin cream in the treatment of 100 cases of chronic hand and foot eczema,” *Journal of Traditional Chinese Medicine*, vol. 50, no. 5, pp. 392, 2009.

[83] J.S. Hu, “Efficacy observation of Binghuang Fule ointment combined with triamcinolone acetonide and econazole ointment in the treatment of perianal and external genitalia eczema,” *Chinese and Foreign Medical Research*, vol. 7, no. 7, pp. 81-82, 2009.

[84] J.S. Xu, “Efficacy observation of Binghuang Fule ointment combined with mometasone furoate cream in the treatment of hand eczema,” *Chinese Journal of Hydroelectricity Medicine*, vol. 18, no. 01, pp. 25-26, 2006.

[85] W.Q. Huang, T.L. Chen, and F.X. Fan, “Clinical efficacy observation of Wudai ointment in the treatment of chronic eczema,” *China Medical Abstracts of Dermatology*, vol. 29, no. 4, pp. 203,205, 2012.

[86] Y. Liu, X.Q. Sun, J. Yang et al., “Clinical efficacy of Wudai ointment in the treatment of rhagadia manus and pedis eczema,” *Chinese Journal of Coal Industry Medicine*, vol. 14, no. 9, pp. 1284-1285, 2011.

[87] X.J. Hu and S.X. Wang, “Comparison of Wudai ointment with triamcinolone acetonide and econazole cream in the treatment of four kinds of skin diseases,” *Clinical Journal of Chinese Medicine*, vol. 2, no. 2, pp. 63-64, 2010.

[88] S.H. Zheng, “Efficacy observation of Wudai ointment in the treatment of hand eczema,” *Hubei Journal of Traditional Chinese Medicine*, vol. 31, no. 9, pp. 54, 2009.

[89] W.H. Wang, B.L. Xu, and Y. Luan, “Wudai ointment in the treatment of 60 cases of neurodermatitis,” *Hunan Journal of Traditional Chinese Medicine*, vol. 24, no. 2, pp. 71-72, 2008.

[90] G.J. Zhang, “Efficacy observation of Wudai ointment in the treatment of neurodermatitis,” *Journal of New Chinese Medicine*, vol. 44, no. 8, pp. 110-111, 2012.

[91] S.W. Ding, P. Dai, and F.M. Hu, “Wudai ointment combined with clobetasol propionate cream in the treatment of 38 cases of eczema,” *Jiangxi Journal of Traditional Chinese Medicine*, vol. 42, no. 11, pp. 23-24, 2011.

[92] H. Tian, “Clinical experience of Wudai ointment combined with mometasone furoate cream in the treatment of chronic eczema,” *Word Latest Medicine Information*, vol. 15, no. 101, pp. 142,144, 2015.

[93] J.H. Liu, B.R. Huang, W.F. Huang et al., “Efficacy observation of halometasone cream combined with Paeonol ointment in the treatment of infantile eczema,” *China Medical Herald*, vol. 7, no. 24, pp. 48-49, 2010.

[94] X.S. Li, “Efficacy observation of Paeonol ointment in the treatment of chronic hand eczema,” *Biotech World*, no. 05, pp. 68-69, 2014.

[95] Z.J. Liang, “Efficacy observation of Paeonol ointment combined with hydrocortisone butyrate cream in the treatment of facial eczema in infants,” *Medical Aesthetics and Cosmetology*, no. 2, pp. 322, 2015.

[96] Z.Y. Wang, “Efficacy observation of Paeonol ointment combined with hydrocortisone butyrate cream in the treatment of facial eczema in infants,” *Chinese Journal of Clinical Rational Drug Use*, vol. 6, no. 11, pp. 49, 2013.

[97] W.B. Wei, “Efficacy observation of traditional Chinese medicine combined with western medicine for infantile eczema,” *Journal of Snake*, vol. 19, no. 4, pp. 268-269, 2007.

[98] L.H. Bao, “Efficacy observation of Paeonol ointment combined with clobetasol propionate cream in the treatment of eczema,” *China Foreign Medical Treatment*, vol. 29, no. 34, pp. 129, 2010.

[99] Q, Qi, “Clinical efficacy observation of Paeonol ointment combined with dexamethasone liniment in the treatment of eczema,” *World Health Digest*, vol. 6, no. 27, pp. 113, 2009.

[100] D.Q. Yang, Y.P. Bai, and Y.D. Liu, “A clinical study on integrated traditional Chinese and western medicine in the treatment of hand eczema in female,” *Journal of Beijing University of Traditional Chinese Medicine*, vol. 28, no. 1, pp. 82-83, 2005.

[101] L. Wu, R.Q. Wu, and Q. Si, “Clinical observation of Pifukang lotion in the treatment of eczema and dermatitis,” *Journal of Inner Mongolia Medical University*, vol. 26, no. 2, pp. 116-117, 2004.

[102] B.G. Yu, G.D. Jiang, and H.Z. Jin, “Pifukang lotion in the treatment of 30 cases of acute and subacute eczema,” *Chinese Journal of Integrated Traditional and Western Medicine*, vol. 19, no. 8, pp. 499, 1999.

[103] M.Y. Li and W.H. Xue, “Efficacy observation of Pifukang lotion in the treatment of 70 cases of acute eczema,” *Journal of Capital Medical University*, vol. 18, no. 1, pp. 92, 1997.

[104] J. Mo, “Efficacy observation of Pifukang lotion combined with hydrocortisone butyrate ointment in the treatment of infantile eczema,” *Modern Journal of Integrated Traditional Chinese and Western Medicine*, vol. 16, no. 14, pp. 1926-1927, 2007.

[105] Y.S. Li and L. Ding, “Efficacy observation of triamcinolone acetonide and econazole cream combined with Pifukang lotion in the treatment of infantile eczema,” *Global Traditional Chinese Medicine*, vol. 7, no. S1, pp. 106-107, 2014.

[106] Y.B. Bai, D.Q. Yang, Y.M. Wang, H. Ju, and M. Chang, “Clinical study of Shuangfujin in the treatment of acute eczema,” *Chinese Journal of Integrated Traditional and Western Medicine*, vol. 27, no. 1, pp. 72-75, 2007.

[107] X.Y. Yuan, W.J. Gu, and G.Q. Zhang, “Efficacy observation of Sophora alopecuroide oil liniment in the treatment of stasis dermatitis,” *Hebei Journal of Traditional Chinese Medicine*, vol. 35, no. 5, pp. 746-747, 2013.

[108] X.Y. Yuan, W.J. Gu, and G.Q. Zhang, “Efficacy observation of Sophora alopecuroide oil liniment in the treatment of nummular eczema,” *Hebei Journal of Traditional Chinese Medicine*, vol. 35, no. 4, pp. 584-585, 2013.

[109] X.Y. Yuan, S.Y. Bi, and G.Q. Zhang, “Efficacy observation of Sophora alopecuroide oil liniment in the treatment of eczema,” *Hebei Medical Journal*, vol. 35, no. 7, pp. 1056-1057, 2013.

[110] X.Y Yuan, W.J. Gu, and G.Q. Zhang, “Clinical observation of Sophora alopecuroide oil liniment in the treatment of neurodermatitis,” *Hebei Journal of Traditional Chinese Medicine*, vol. 35, no. 3, pp. 412-413, 2013.

[111] S.R. Da and F.L. Sun, “Efficacy observation of Sophora alopecuroide oil liniment in the treatment of neurodermatitis,” *Journal of Dermatology and Venereology*, vol. 33, no.3, pp. 160, 2011.

[112] K.F. Gu, “Clinical observation of Sophora alopecuroide oil liniment in the treatment of eczema,” *Chinese Journal of Practical Chinese with Modern Medicine*, vol. 21, no.6, pp. 493-494, 2008.

[113] W.B. Hu, “Observation of the effect of hydrocortisone butyrate cream combined with Erfukang liniment on infant eczema,” *China Tropical Medicine*, vol. 9, no. 12, pp. 2270-2271, 2009.

[114] J.H. Wang, “Clinical observation of combined therapy in the treatment of subacute eczema in infants,” *Modern Journal of Integrated Traditional Chinese and Western Medicine*, vol. 19, no. 27, pp. 3451-3452, 2010.

[115] X.L. Rao, H. Wang, S.N. Lin, Y.W. Wu, and Y.Y. Chen, “Clinical effectiveness of hydrocortisone butyrate cream combined with Erfukang liniment in the treatment of 45 cases of infantile eczema,” *Guide of China Medicine*, vol. 10, no. 33, pp.126-127, 2012.

[116] W.F. Geng and Y. Wang, “Erfukang liniment combined with loratadine syrup in the treatment of infantile eczema,” *Gansu Medical Journal*, vol. 30, no.10, pp. 582-583, 2011.

[117] H.M. Li, “Analysis of the effect of Erfukang liniment for 106 cases of infantile eczema,” *Shandong Medical Journal*, vol. 45, no. 27, pp. 56, 2005.

[118] J.J. Qian, “Comparison Erfukang liniment with hydrocortisone butyrate cream in the treatment of infantile eczema,” *Journal of Dermatology and Venereology*, vol. 28, no. 2, pp. 29, 2006.

[119] Y.E. Hu, “Clinical observation of Qiangyue cream in the treatment of neurodermatitis,” *Pharmacology and Clinics of Chinese Materia Medica*, vol. 31, pp. 6, pp. 195-197, 2015.

[120] X. Zhang, W.G. Sun, and J. Sun, “Clinical study on Qiangyue cream in the treatment of neurodermatitis,” *Chinese Journal of Integrated Traditional and Western Medicine*, vol. 27, no. 12, pp. 1126-1128, 2007.

[121] L.F. Zhang, “Comparison of Qiangyue cream with hydrocortisone butyrate cream in the treatment of facial subacute eczema in infants,” *Journal of Shangdong Medical College*, vol. 37, no. 6, pp. 457-458, 2015.

[122] W.G. Sun and X. Zhang, “Efficacy observation of Qiangyue cream in the treatment of localized neurodermatitis,” *China Journal of Leprosy and Skin Diseases*, vol. 25, no. 6, pp. 480-481, 2009.

[123] H.L. Lou, G.X. Fang, and G.H. Hu, “Efficacy observation of Qiangyue cream and loratadine syrup for 65 cases of atopic dermatitis in children,” *Journal of Pediatrics of Traditional Chinese Medicine*, vol. 11, no. 4, pp. 42-44, 2015.

[124] W.G. Sun and X. Zhang, “Efficacy observation of Qiangyue cream with hydrocortisone butyrate ointment in the treatment of eczema in children,” *Journal of Clinical Dermatology*, vol. 38, no. 12, pp. 804-805, 2009.

[125] J.J. Lyu, “Efficacy observation of Meilu Xiaocuo ointment combined with mizolastine tablet in the treatment of facial dermatitis,” *Chinese Practical Medicine*. vol. 5, no. 3, pp. 174, 2010.

[126] A.M. Shu, “Efficacy observation of Meilu Xiaocuo ointment in the treatment of facial seborrheic dermatitis,” *Practical Clinical Journal of Intergrated Traditional Chinese and Western Medicine*, vol. 9, no. 6, pp. 39-40, 2009.

[127] M.H. Yu, “Efficacy observation of Mayinglong musk hemorrhoid cream in the treatment of perianal eczema,” *China Practical Medical*, vol. 8, no. 8, pp. 159-160, 2013.

[128] X.D. Luo, “Efficacy observation of Geranium ointment combined with desonide cream in the treatment of eczema in children,” *Journal of Dermatology and Venereology*, vol. 40, no. 2, pp. 229-230, 2018.

[129] D. Du, “Efficacy of Chushi Zhiyang lotion combined with desonide cream in the treatment of eczema,” *Chinese Journal of Dermatovenerology of Integrated Traditional and Western Medicine*, vol. 13, no. 1, pp. 31-32, 2014.

[130] M.F. Yang, “Integrated traditional Chinese and western medicine in the treatment of 43 cases of perianal eczema,” *Journal of External Therapy of Traditional Chinese Medicine*, vol. 20, no. 4, pp. 31, 2011.

[131] Z. Yu and N. Yu, “Clinical observation between Jieeryin lotion and topical corticosteroids in the treatment of neurodermatitis,” *Journal of Ningxia Medical College*, vol. 18, no. 4, pp. 41,58, 2016.

[132] H. Gao, J. Chen, and J.Y. Li, “Efficacy of Xiaofeng Zhiyang granule in the treatment of acute eczema,” *China Journal of Leprosy and Skin Diseases*, vol. 27, no. 4, pp. 239, 2011.

[133] Y. Jiang, “Clinical Efficacy of Xiaofeng Zhiyang granule in the treatment of eczema,” *China Health Standard Management*, vol. 7, no. 4, pp. 141-142, 2016.

[134] C.Y. Liu, “Clinical observation of Xiaofeng Zhiyang granule combined with ebastine tablet for 40 cases of chronic eczema,” *Yunnan Journal of Traditional Chinese Medicine and Materia Medica*, vol. 36, no. 1, pp. 48-49, 2015.

[135] X. Wang, B. Wang, J.G. Wang, and H.M. Xu, “Efficacy of Xiaofeng Zhiyang granule combined with cetirizine hydrochloride tablet in the treatment of eczema and dermatitis,” *Chinese Journal of Trauma and Disability Medicine*, vol. 21, no. 6, pp. 218-219, 2013.

[136] W.G. Cai, “Integrated traditional Chinese and western medicine in the treatment of 60 cases of chronic eczema,” *Tibetan Medicine*, vol. 31, no. 3, pp. 18-20, 2010.

[137] R. Zhang, J. Jia, T.Y. Zheng et al., “Clinical observation of Piminxiao capsule in the treatment of damp-heat eczema,” *Yi Yao Jie*, vol. 12, pp. 33, 2019.

[138] D.B. Su and W.L. Sun, “Efficacy of Piminxiao capsule combined with levocetirizine hydrochloride tablet for 132 cases of acute eczema,” *Nei Mongol Journal of Traditional Chinese Medicine*, vol. 32, no. 27, pp. 3, 2013.

[139] J.X. Lyu, “Analysis of clinical efficacy of Baixuanxiatare tablet in the treatment of seborrheic dermatitis,” *China Continuing Medical Education*, vol. 9, no. 33, pp. 103-104, 2017.

[140] P.J. Wang, Y.H. Ye, and F.Q. Xiang, “Clinical observation of Baixuanxiatare tablet in the treatment of chronic eczema,” *Chinese Community Doctors*, vol. 13, no. 26, pp. 165-166, 2011.

[141] J. Bai, “Clinical observation of Phellodendron bark capsule in the treatment of 90 cases of eczema,” *Journal of Practical Medical Techniques*, vol. 15, no. 27, pp. 3704-3705, 2008.

[142] X. Zhang, “Phellodendron bark capsule in the treatment of sixty patients with eczema,” *Journal of Traditional Chinese Medicine*, vol. 44, no. 1, pp. 57, 2003.

[143] T. Zhao, W.L. Liu, P. Wu et al., “A randomized, placebo-controlled study on Fangfeng Tongsheng granule in the treatment of subacute eczema,” *China Journal of Chinese Materia Medica*, vol. 40, no. 7, pp. 1415-1418, 2015.

[144] Y. Qiu, “Effects of Sophora flavescens capsules on serum Th1 and Th2 cytokine levels in patients with damp-heat chronic eczema,” *Medical Journal of Chinese people's Health*, vol. 30, no. 5, pp. 80-82, 2018.
