# Supplementary material for: Reporting Quality of Randomized Controlled Trials for the Treatment of Eczema with Chinese Patent Medicine Based on the CONSORT-CHM Formulas 2017
Source: Evid Based Complement Alternat Med. 2020 Sep 14;2020:2949125. doi: 10.1155/2020/2949125 (PMC7512083; doi:10.1155/2020/2949125)
Supplement: Supplementary Materials — Supplementary Table 1: checklist of items for the CONSORT-CHM formulas 2017. Supplementary Table 2: the list of Chinese patent medicine with the indication for eczema. Supplementary File 1: the list of 144 randomized controlled trials on Chinese patent medicine for eczema. Supplementary Table 3: the characteristics of 144 randomized controlled trials on Chinese patent medicine for eczema. Supplementary Table 4: the score of each item for 144 randomized controlled trials based on the CONSORT-CHM formulas 2017. [file 2949125.f1.zip › 2949125.f1/Supplementary Table 1 Checklist of items for CONSORT-CHM formulas 2017 (1).docx]

Supplementary Table 1: Checklist of items for the CONSORT-CHM formulas 2017.

| **Section/Topic** | **Item**  **number** | **Standard CONSORT Checklist Item** | **Extension for CONSORT-CHM formulas 2017** | **Page**  **Number** |
| --- | --- | --- | --- | --- |
| **Title, abstract,**  **and key words** |  |  |  |  |
| Title | 1a | Identification as a randomized trial in the title | Statement of whether the trial targets a TCM Pattern, a Western medicine–defined disease, or a Western medicine–defined disease with a specific TCM Pattern, if applicable |  |
| Abstract | 1b | Structured summary of trial design, methods, results, and conclusions (for specific guidance see CONSORT for abstracts) | Illustration of the name and form of the formula  used, and the TCM Pattern applied, if applicable |  |
| Keywords | 1c |  | Determination of appropriate keywords, including “Chinese herbal medicine formula” and “randomized controlled trial” |  |
| **Introduction** |  |  |  |  |
| Background | 2a | Scientific background and explanation of rationale | Statement with biomedical science approaches  and/or TCM approaches |  |
| Objectives | 2b | Specific objective or hypotheses | Statement of whether the formula targets a Western medicine–defined disease, a TCM Pattern, or a Western medicine–defined disease with a specific TCM Pattern |  |
| **Methods** |  |  |  |  |
| Trial design | 3a | Description of trial design (such as parallel and factorial) including allocation ratio |  |  |
|  | 3b | Important changes to methods after trial commencement (such as eligibility criteria) with reasons |  |  |

Supplementary Table 1-continued

| **Section/Topic** | **Item**  **number** | **Standard CONSORT Checklist Item** | **Extension for CONSORT-CHM formulas 2017** | **Page**  **Number** |
| --- | --- | --- | --- | --- |
| Participants | 4a | Eligibility criteria for participants | Statement of whether participants with a specific TCM Pattern were recruited, in terms of 1) diagnostic criteria and 2) inclusion and exclusion criteria. All criteria used should be universally recognized, or reference given to where detailed  explanation can be found. |  |
|  | 4b | Settings and locations where the data were collected |  |  |
| Interventions | 5 | The interventions for each group with sufficient details to allow replication, including how and when they were actually administered | Description(s) for different types of formulas should  include the following:  **5a. For fixed CHM formulas**  1. Name, source, and dosage form (e.g., decoctions, granules, powders)  2. Name, source, processing method, and dosage of each medical substance. Names of substances should be presented in at least 2 languages: Chinese (Pinyin), Latin, or English. Names of the parts of the substances used should be specified.  3. Authentication method of each ingredient and how, when, where, and by whom it was conducted; statement of whether any voucher specimen was retained, and if so, where they were  kept and whether they are accessible  4. Principles, rationale, and interpretation of forming the formula  5. Reference(s) as to the efficacy of the formula, if any  6. Pharmacologic study results of the formula, if any  7. Production method of the formula, if any |  |

Supplementary Table 1-continued

| **Section/Topic** | **Item**  **number** | **Standard CONSORT Checklist Item** | **Extension for CONSORT-CHM formulas 2017** | **Page**  **Number** |
| --- | --- | --- | --- | --- |
|  |  |  | 8. Quality control of each ingredient and of the product of the formula, if any. This would include any quantitative and/or qualitative testing method(s); when, where, how, and by whom these tests were conducted; whether the original data  and samples were kept, and, if so, whether they are accessible.  9. Safety assessment of the formula, including tests for heavy metals and toxic elements, pesticide residues, microbial limit, and acute/chronic toxicity, if any. If yes, it should be stated when, where, how, and by whom these tests were conducted; if the original data and samples were kept; and, if so, whether they are accessible.  10. Dosage of the formula, and how the dosage was determined  11. Administration route (e.g., oral, external)  **5b. For individualized CHM formulas**  1. See recommendations 5a 1–11  2. Additional information: how, when, and by  whom the formula was modified  **5c. For patent proprietary CHM formulas**  1. Reference to publicly available materials, such as pharmacopeia, for the details about the composition, dosage, efficacy, safety, and quality control of the formula  2. Illustration of the details of the formula, namely  1) the proprietary product name (i.e., brand name), 2) name of manufacturer, 3) lot number, 4) production date and expiry date, |  |

Supplementary Table 1-continued

| **Section/Topic** | **Item**  **number** | **Standard CONSORT Checklist Item** | **Extension for CONSORT-CHM formulas 2017** | **Page**  **Number** |
| --- | --- | --- | --- | --- |
|  |  |  | 5) name and percentage of added materials, and 6) whether any additional quality control measures were conducted  3. Statement of whether the patent proprietary  formula used in the trial is for a condition that is identical to the publicly available reference  **5d. Control groups**  Placebo control  1. Name and amount of each ingredient  2. Description of the similarity of placebo with the intervention (e.g., color, smell, taste, appearance, packaging)  3. Quality control and safety assessment, if any  4. Administration route, regimen, and dosage  5. Production information: where, when, how, and by whom the placebo was produced  Active control  1. If a CHM formula was used, see recommendations 5a–5c  2. If a chemical drug was used, see item 5 of the CONSORT Statement |  |
| Outcomes | 6a | Completely defined prespecified primary and secondary outcome measures, including how and when they were assessed | Illustration of outcome measures with Pattern in detail |  |
|  | 6b | Any changes to trial outcomes after the trial commenced, with reasons |  |  |

Supplementary Table 1-continued

| **Section/Topic** | **Item**  **number** | **Standard CONSORT Checklist Item** | **Extension for CONSORT-CHM formulas 2017** | **Page**  **Number** |
| --- | --- | --- | --- | --- |
| Sample size | 7a | How sample size was determined |  |  |
|  | 7b | When applicable, explanation of any interim analyses and stopping guidelines |  |  |
| Randomization |  |  |  |  |
| Sequence generation | 8a | Methods used to generate the random allocation sequence |  |  |
|  | 8b | Type of randomization; details of any restrictions (such as blocking and block size) |  |  |
| Allocation concealment  mechanism | 9 | Mechanism used to implement the random allocation sequence (such as sequentially numbered containers), describing any steps taken to conceal the sequence until interventions were assigned |  |  |
| Implementation | 10 | Who generated the random allocation sequence, who enrolled participants, and who assigned participants to interventions |  |  |
| Blinding | 11a | If done, who was blinded after assignment to interventions (eg, participants, care providers, and those assessing outcomes) and how |  |  |
|  | 11b | If relevant, description of the similarity of interventions |  |  |

Supplementary Table 1-continued

| **Section/Topic** | **Item**  **number** | **Standard CONSORT Checklist Item** | **Extension for CONSORT-CHM formulas 2017** | **Page**  **Number** |
| --- | --- | --- | --- | --- |
| Statistical methods | 12a | Statistical methods used to compare groups |  |  |
|  | 12b | Methods for additional analyses, such as analyses and adjusted analyses |  |  |
| **Results** |  |  |  |  |
| Participant flow | 13a | For each, the numbers of participants who was were randomly assigned, received intended treatment, and were analyzed for the primary outcome group |  |  |
|  | 13b | For each group, loss and exclusions after randomization, together with reasons |  |  |
| Recruitment | 14a | Dates defining the periods of recruitment and follow-up |  |  |
|  | 14b | Why the trial ended or was stopped |  |  |
| Baseline data | 15 | A table showing baseline demographic and clinical characteristics for each group |  |  |
| Number analyzed | 16 | For each group, number of participants (denominator) included in each analysis and whether the analysis was by original assigned groups |  |  |
| Outcomes  and estimation | 17a | For each primary and secondary outcome, results for each group, and the estimated effect size and precision (such as 95% confidence interval) |  |  |

Supplementary Table 1-continued

| **Section/Topic** | **Item**  **number** | **Standard CONSORT Checklist Item** | **Extension for CONSORT-CHM formulas 2017** | **Page**  **Number** |
| --- | --- | --- | --- | --- |
|  | 17b | For binary outcomes, presentation of both absolutes and relative effect sizes is recommended |  |  |
| Ancillary analyses | 18 | Results of any other analyses performed, including subgroup analyses and adjusted analyses, distinguishing prespecified from exploratory |  |  |
| Harms | 19 | All important harms or unintended effects in each group (for specific guidance see CONSORT for harms) |  |  |
| **Discussion** |  |  |  |  |
| Limitations | 20 | Trial limitations, addressing sources of potential bias, imprecision, and, if relevant, multiplicity of analyses |  |  |
| Generalizability | 21 | Generalizability (external validity, applicability) of the trial findings. | Discussion of how the formula works on different TCM Patterns or diseases |  |
| Interpretation | 22 | Interpretation consistent with results, balancing benefits and harms, and considering other relevant evidence. | Interpretation with TCM theory |  |
| **Other information** |  |  |  |  |
| Registration | 23 | Registration number and name of trial registry |  |  |
| Protocol | 24 | Location where the full trial protocol can be accessed, if available |  |  |
| Funding | 25 | Sources of funding and other support (such as supply of drugs), roles of funders |  |  |

CHM: Chinese herbal medicine; CONSORT: Consolidated Standard of Reporting Trials; TCM: traditional Chinese medicine
