# Supplementary material for: Reporting Quality of Randomized Controlled Trials for the Treatment of Eczema with Chinese Patent Medicine Based on the CONSORT-CHM Formulas 2017
Source: Evid Based Complement Alternat Med. 2020 Sep 14;2020:2949125. doi: 10.1155/2020/2949125 (PMC7512083; doi:10.1155/2020/2949125)
Supplement: Supplementary Materials — Supplementary Table 1: checklist of items for the CONSORT-CHM formulas 2017. Supplementary Table 2: the list of Chinese patent medicine with the indication for eczema. Supplementary File 1: the list of 144 randomized controlled trials on Chinese patent medicine for eczema. Supplementary Table 3: the characteristics of 144 randomized controlled trials on Chinese patent medicine for eczema. Supplementary Table 4: the score of each item for 144 randomized controlled trials based on the CONSORT-CHM formulas 2017. [file 2949125.f1.zip › 2949125.f1/Supplementary Table 2 The list of Chinese patent medicine with the indication for eczema (1).docx]

Supplementary Table 2: The list of Chinese patent medicine with the indication for eczema.

| No. | Topical Chinese patent medicine | No. | Oral Chinese patent medicine |
| --- | --- | --- | --- |
| 1 | Qingpeng ointment | 1 | Baixuanxiatare tablet |
| 2 | Chushi Zhiyang ointment | 2 | Baixuanxiatare dispersible tablet |
| 3 | Binghuang Fule ointment | 3 | Baixuanxiatare capsule |
| 4 | Paeonol ointment | 4 | Phellodendron bark tablet |
| 5 | Wudai ointment | 5 | Phellodendron bark capsule |
| 6 | Geranium ointment | 6 | Qingre Sanjie tablet |
| 7 | Compound Luotuopengzi ointment | 7 | Qingre Sanjie capsule |
| 8 | Shenhuang ointment | 8 | Sophora flavescens tablet |
| 9 | Meilu Xiaocuo ointment | 9 | Sophora flavescens capsule |
| 10 | Qiangyue cream | 10 | Pifubing Xuedu tablet |
| 11 | Mayinglong musk hemorrhoid cream | 11 | Pifubing Xuedu pill |
| 12 | Qishenlian eczema cream | 12 | Compound Zhenzhu Anchuang tablet |
| 13 | Pifukang lotion | 13 | Compound Zhenzhu Anchuang capsule |
| 14 | Jieeryin lotion | 14 | Fangfeng Tongsheng tablet |
| 15 | Shenbai lotion | 15 | Fangfeng Tongsheng granule |
| 16 | Jieshen lotion | 16 | Danggui Kushen pill |
| 17 | Qingbai Jieshen lotion | 17 | Xiaofeng Zhiyang granule |
| 18 | Shuangzishen lotion | 18 | Piminxiao capsule |
| 19 | Chushi Zhiyang lotion | 19 | Jiedu capusle |
| 20 | Huangpu Jiefu lotion | 20 | Andrographis paniculata capsule |
| 21 | Fujingkang lotion | 21 | Wuhuacha granule |
| 22 | Fuji lotion | 22 | Liangxue Qufeng syrup |
| 23 | Shaoshang Fukang lotion | 23 | Yaozhi Guiling jelly |
| 24 | Sophora alopecuroide oil liniment | 24 | Xixian pill |
| 25 | Erfukang liniment | 25 | Xixian Fengshi pill |
| 26 | Fukang liniment | 26 | Qingdajiang pill |
| 27 | Shiyang aerosol | 27 | Urticaria pill |
| 28 | Shangkeling spray | 28 | Shibawei Dangshen pill |
| 29 | Keyangmin agent | 29 | Shibawei Ouqu pill |
| 30 | Babao Wudan ink | 30 | Shibawei Ouquzhenbao pill |
| 31 | Zhiyang tincture | 31 | Shiwei Ruxiang pill |
| 32 | Kangfu tincture | 32 | Shiwei Ruxiang powder |
| 33 | Shufu Zhiyang tincture | 33 | Shiwei Ruxiang capsule |
| 34 | Songhua powder |  |  |
| 35 | Miliaria powder |  |  |
| 36 | Qingge powder |  |  |
| 37 | Huangshuichuang powder |  |  |
