# Supplementary material for: Reporting Quality of Randomized Controlled Trials for the Treatment of Eczema with Chinese Patent Medicine Based on the CONSORT-CHM Formulas 2017
Source: Evid Based Complement Alternat Med. 2020 Sep 14;2020:2949125. doi: 10.1155/2020/2949125 (PMC7512083; doi:10.1155/2020/2949125)
Supplement: Supplementary Materials — Supplementary Table 1: checklist of items for the CONSORT-CHM formulas 2017. Supplementary Table 2: the list of Chinese patent medicine with the indication for eczema. Supplementary File 1: the list of 144 randomized controlled trials on Chinese patent medicine for eczema. Supplementary Table 3: the characteristics of 144 randomized controlled trials on Chinese patent medicine for eczema. Supplementary Table 4: the score of each item for 144 randomized controlled trials based on the CONSORT-CHM formulas 2017. [file 2949125.f1.zip › 2949125.f1/Supplementary Table 3 The characteristics of 144 randomized controlled trials on Chinese patent medicine for eczema.docx]

Supplementary Table 3: The characteristics of 144 randomized controlled trials on Chinese patent medicine for eczema

| First author | Sample size  (Male/Female) | Age Range or  Mean Age (year) | Intervention | Treatment  duration | Outcomes | Results |
| --- | --- | --- | --- | --- | --- | --- |
| Yan Li^#^  2019 | E: 30(15/15)  C: 30(17/13) | E: 26-72  C: 20-74 | E: Qingpeng ointment (b.i.d)  C: Sham ointment (b.i.d) | 2 weeks | ①②③⑤ | (1A) (2A) (3A) |
| Gang Wang  2018 | E: 60(35/25)  C: 60(33/27) | E: 2-11  C: 2-12 | E: Qingpeng ointment (b.i.d)  C: Vaseline ointment (b.i.d) | 2 weeks | ①②⑤ | (1A) (2A) |
| Junbi Zhang  2017 | E: 34(18/16)  C: 33(15/18) | E: 2-12  C: 3-12 | E: Qingpeng ointment (b.i.d)  C: Vaseline ointment (b.i.d) | 2 weeks | ①⑤ | (1A) |
| Yuanyao She  2016 | E: 33(16/17)  C: 32(16/16) | E: 2-12  C: 2-12 | E: Qingpeng ointment (b.i.d)  C: Vaseline ointment (b.i.d) | 2 weeks | ①②⑤ | (1A) (2A) |
| Chunzhi Dong  2014 | E: 25(16/17)  C: 25(14/11) | E: 25-67  C: 23-71 | E: Qingpeng ointment (b.i.d)  C: Vaseline ointment (b.i.d) | 4 weeks | ① | (1A) |
| Jia Zeng  2013 | E: 38(20/18)  C: 34(16/18) | E: 20-63  C: 20-63 | E: Qingpeng ointment (b.i.d)  C: Vaseline ointment (b.i.d) | 4 weeks | ①②⑤ | (1A) (2A) |
| Hui Tang^*^  2011 | E: 159(70/89)  C: 76(26/50) | E: 42.34  C: 42.14 | E: Qingpeng ointment (b.i.d)  C: Sham ointment (b.i.d) | 3 weeks | ①②⑤ | (1A) (2A) |
| Shaojun Chen  2010 | E: 43(17/26)  C: 40(19/21) | E: 29-68  C: 31-69 | E: Qingpeng ointment (b.i.d)  C: Vaseline ointment (b.i.d) | 4 weeks | ①⑤ | (1A) |
| Shu Li  2019 | E: 40  C: 40 | E: 21-68  C: 22-68 | E: Qingpeng ointment (b.i.d)  C: 0.1% Tacrolimus ointment (b.i.d) | 4 weeks | ①⑤ | (1C) |
| Cuisong Zhang  2018 | E: 28  C: 29 | E: 29.5  C: 29.38 | E: Qingpeng ointment (b.i.d)  C: 0.1% Tacrolimus ointment (b.i.d) | 4 weeks | ①②⑤ | (1C) (2C) |
| Yan Li^*^  2017 | E: 223(110/113)  C: 203(112/91) | E: 2-16  C: 2-16 | E: Qingpeng ointment (b.i.d)  C: 0.03% Tacrolimus ointment (b.i.d) | 2 weeks | ①②③⑤ | (1B) (2B)  (3B) |
| Kai Tang  2017 | E: 56(35/21)  C: 56(37/19) | E: 22-64  C: 21-63 | E: Qingpeng ointment (t.i.d)  C: Triamcinolone acetonide and econazole ointment (b.i.d) | 2 weeks | ①⑤ | (1A) |
| Shuhua Song  2017 | E: 44(21/23)  C: 43(24/19) | E: 6.4  C: 5.8 | E: Qingpeng ointment (b.i.d)  C: Hydrocortisone butyrate cream (b.i.d) | 4 weeks | ①③⑤ | (1B) (3C) |
| Lingbin Kong  2017 | E: 60(26/34)  C: 60(27/33) | E: 45.5  C: 42.3 | E: Qingpeng ointment (t.i.d)  C: Hydrocortisone butyrate cream (b.i.d) | 2 weeks | ①⑤ | (1A) |
| Jiling Zhang  2016 | E: 30(19/12)  C: 30(16/14) | E: 20-62  C: 22-66 | E: Qingpeng ointment (t.i.d)  C: Triamcinolone acetonide and econazole ointment (b.i.d) | 2 weeks | ① | (1B) |

Supplementary Table 3: The characteristics of 144 randomized clinical trials on Chinese patent medicine for eczema (continued)

| First author | Sample size  (Male/Female) | Age Range or  Mean Age (year) | Intervention | Treatment  duration | Outcomes | Results |
| --- | --- | --- | --- | --- | --- | --- |
| Jing Han  2016 | E: 40(21/19)  C: 40(22/18) | E: 20-78  C: 21-76 | E: Qingpeng ointment (t.i.d)  C: Hydrocortisone butyrate cream (b.i.d) | 2 weeks | ①⑤ | (1A) |
| Lanxin Zhang  2016 | E: 33(15/18)  C: 31(14/17) | E: 18-60  C: 18-60 | E: Qingpeng ointment (b.i.d)  C: Hydrocortisone butyrate cream (b.i.d) | 4 weeks | ①⑤ | (1B) |
| Hu Yang  2016 | E: 110(62/48)  C: 110(50/60) | E: 18-64  C: 17-58 | E: Qingpeng ointment (t.i.d)  C: Hydrocortisone butyrate ointment (b.i.d) | 2 weeks | ①②⑤ | (1B) (2B) |
| Suwei Tang  2016 | E: 26 (14/12)  C: 29 (15/14) | E: 47.15  C: 45.69 | E: Qingpeng ointment (b.i.d) + Ebastine tablet (10mg/d)  C: Triamcinolone acetonide and urea cream (b.i.d) + Ebastine tablet (10mg/d) | 2 weeks | ①③⑤ | (1B) (3C) |
| Jinzhu Guo  2015 | E: 26(11/15)  C: 27(13/14) | E: 3.96  C: 5.59 | E: Qingpeng ointment (b.i.d)  C: 0.03% Tacrolimus ointment (b.i.d) | 1 week | ①②③⑤ | (1B) (2B)  (3B) |
| Jia Guo  2015 | E: 40(16/24)  C: 40(18/22) | E: 15-70  C: 16-68 | E: Qingpeng ointment (b.i.d)  C: Halometasone cream (b.i.d) | 4 weeks | ①⑤ | (1B) |
| Shen Yi  2015 | E: 45(23/22)  C: 45(21/24) | E: 1-14  C: 1-13 | E: Qingpeng ointment (b.i.d)  C: Mometasone furoate cream (b.i.d) | 2 weeks | ①②⑤ | (1B) (2B) |
| Ming Gao  2015 | E: 43(20/23)  C: 44(19/25) | E: 26  C: 27 | E: Qingpeng ointment (b.i.d)  C: Triamcinolone acetonide and miconazole nitrate and neomycin sulfate cream (b.i.d) | 2 weeks | ①⑤ | (1B) |
| Danyun Huang  2014 | E: 40(13/27)  C: 40(11/29) | E: 38.36  C: 38.26 | E: Qingpeng ointment (b.i.d)  C: Hydrocortisone butyrate cream (b.i.d) | 3 weeks | ①③⑤ | (1B) (3B) |
| Wenjun Hou  2014 | E: 34  C: 37 | E: NA  C: NA | E: Qingpeng ointment (b.i.d)  C: Hydrocortisone butyrate cream (b.i.d) | 3 weeks | ①⑤ | (1C) |
| Zigang Zhao  2011 | E: 33  C: 32 | E: NA  C: NA | E: Qingpeng ointment (b.i.d)  C: Hydrocortisone cream (b.i.d) | 4 weeks | ①②⑤ | (1B) (2B) |
| Hua Wang  2011 | E: 128 (63/65)  C: 121 (58/63) | E: 18-65  C: 16-60 | E: Qingpeng ointment (t.i.d)  C: Hydrocortisone butyrate ointment (b.i.d) | 2 weeks | ①②⑤ | (1B) (2B) |
| Wenhua Zeng  2011 | E: 22(10/12)  C: 20(10/10) | E: 52.09  C: 43.00 | E: Qingpeng ointment (b.i.d)  C: Hydrocortisone butyrate ointment (b.i.d) | 4 weeks | ①⑤ | (1B) |
| Jingbing Li  2011 | E: 38  C: 29 | E: 42-78  C: 42-78 | E: Qingpeng ointment (b.i.d to t.i.d)  C: Halometasone cream (b.i.d to. t.i.d) | 4 weeks | ①④ | (1A) (4B) |
| Zhanxue Sun  2010 | E: 68(33/35)  C: 69(34/35) | E: 39.15  C: 37.43 | E: Qingpeng ointment (b.i.d)  C: Hydrocortisone butyrate cream (b.i.d) | 2 weeks | ①③④ | (1B) (3B)  (4A) |

Supplementary Table 3: The characteristics of 144 randomized clinical trials on Chinese patent medicine for eczema (continued)

| First author | Sample size  (Male/Female) | Age Range or  Mean Age (year) | Intervention | Treatment  duration | Outcomes | Results |
| --- | --- | --- | --- | --- | --- | --- |
| Li Gao  2015 | E: 48(26/22)  C: 48(25/23) | E: 19-58  C: 18-60 | E: Qingpeng ointment (b.i.d) + Ebastine tablet (10mg/d)  C: Ebastine tablet (10mg/d) | 4 weeks | ① | (1A) |
| Yang Yang  2018 | E: 37(18/19)  C:37(17/20) | E: 4-10  C: 4-9 | E: Qingpeng ointment (b.i.d) + Desonide ointment (b.i.d)  C: Desonide ointment (b.i.d) | 4 weeks | ①②③⑤ | (1A) (2A) (3A) |
| Jiaqi Jiang  2017 | E: 50(19/78)  C: 50(30/20) | E: 19-78  C: 20-81 | E: Qingpeng ointment (b.i.d) + Mometasone furoate cream (b.i.d)  C: Mometasone furoate cream (b.i.d) | 2 weeks | ①③⑤ | (1A) (3A) |
| Yanfeng He  2016 | E: 48(24/24)  C: 48(25/23) | E: 0.13-1  C: 0.11-1 | E: Qingpeng ointment (b.i.d) + Hydrocortisone butyrate cream (b.i.d)  C: Hydrocortisone butyrate cream (b.i.d) | 2 weeks | ①⑤ | (1A) |
| Yaqin Li  2016 | E: 75(40/35)  C: 75(41/34) | E: 20-53  C: 21-51 | E: Qingpeng ointment (t.i.d) + Halometasone cream (b.i.d)  C: Halometasone cream (b.i.d) | 2 weeks | ①⑤ | (1A) |
| Xiaohong Wang  2015 | E: 54(23/31)  C: 44(19/25) | E: 18-70  C: 18-70 | E: Qingpeng ointment (b.i.d) + Hydrocortisone butyrate cream (b.i.d)  C: Hydrocortisone butyrate cream (b.i.d) | 2 weeks | ①②⑤ | (1A) (2A) |
| Lin Zhou  2011 | E: 63  C: 63 | E: 15-70  C: 15-70 | E: Qingpeng ointment (b.i.d) + Halometasone cream (b.i.d)  C: Halometasone cream (b.i.d) | 2 weeks | ①②⑤ | (1A) (2A) |
| Xiaoxiang Zhai  2011 | E: 32(13/19)  C: 32(12/20) | E: 16-64  C: 16-64 | E: Qingpeng ointment (b.i.d) + Fluticasone propionate cream (b.i.d)  C: Fluticasone propionate cream (b.i.d) | 2 weeks | ①②⑤ | (1A) (2A) |
| Guanghui Peng  2011 | E: 44(16/28)  C: 41(14/27) | E: 19-63  C: 18-64 | E: Qingpeng ointment (b.i.d) + Triamcinolone acetonide and econazole cream (b.i.d)  C: Triamcinolone acetonide and econazole cream (b.i.d) | 4 weeks | ①②⑤ | (1A) (2A) |
| Liqing Yu  2018 | E: 72  C: 70 | E: NA  C: NA | E: Qingpeng ointment (b.i.d) + Desonide cream (b.i.d) (Week 1 and Week 2)  Qingpeng ointment (b.i.d) (Week 3 and Week 4)  C: Desonide cream (b.i.d) (Week 1, Week 2, Week 3 and Week4) | 4 weeks | ①⑤ | (1B) |
| Yunfeng Zhang  2014 | E: 43  C: 32 | E: 16-80  C: 16-80 | E: Qingpeng ointment (b.i.d) + Hydrocortisone butyrate cream (q.d) (Week 1)  Qingpeng ointment (b.i.d) (Week 2 and Week 3)  C: Hydrocortisone butyrate cream (b.i.d) (Week 1, Week 2 and Week 3) | 3 weeks | ①⑤ | (1B) |
| Yanru Liu  2014 | E: 40(21/19)  C: 42(21/21) | E: 21-70  C: 19-72 | E: Qingpeng ointment (q.d) + Halometasone cream (b.i.d) (Week 1)  Qingpeng ointment (b.i.d) + Halometasone cream (q.d) (Week 2 and Week4)  Qingpeng ointment (b.i.d) (Week4)  C: Halometasone cream (b.i.d) (Week 1)  Halometasone cream (q.d) (Week 2, Week 3 and Week 4) | 4 weeks | ①⑤ | (1B) |

Supplementary Table 3: The characteristics of 144 randomized clinical trials on Chinese patent medicine for eczema (continued)

| First author | Sample size  (Male/Female) | Age Range or  Mean Age (year) | Intervention | Treatment  duration | Outcomes | Results |
| --- | --- | --- | --- | --- | --- | --- |
| Ruizhi Teng  2013 | E: 63(37/26)  C: 40(19/21) | E: 29-68  C: 31-69 | E: Qingping ointment (b.i.d) + Hydrocortisone butyrate cream (b.i.d)  (Day 1 to Day 10)  Qingping ointment (b.i.d) (Day 11 to Day 21)  C: Hydrocortisone butyrate cream (b.i.d) (Day 1 to Day 21) | 3 weeks | ①⑤ | (1A) |
| Jianqin Li  2012 | E: 30(16/14)  C: 32(16/16) | E: 21-72  C: 18-75 | E: Qingpeng ointment (q.d) + Fluticasone propionate cream (b.i.d) (Week 1)  Qingpeng ointment (b.i.d) + Fluticasone propionate cream (q.d) (Week 2)  Qingpeng ointment (b.i.d) (Week 3 and Week4)  C: Fluticasone propionate cream (b.i.d) (Week 1)  Fluticasone propionate cream (q.d) (Week 2, Week 3 and Week4) | 4 weeks | ①⑤ | (1B) |
| Jingwen Zheng  2012 | E: 44(16/28)  C: 41(13/28) | E: 18-72  C: 21-65 | E: Qingpeng ointment (q.d) + Mometasone furoate cream (q.d) (Day 1 to Day 7)  Qingpeng ointment (b.i.d) (Day 8 to Day 12)  Mometasone furoate cream (q.d) (Day 13 and Day 14)  Qingpeng ointment (b.i.d) (Day 15 to Day 21)  C: Mometasone furoate cream (q.d) (Day 1 to Day 21) | 3 weeks | ①②③⑤ | (1B) (2A)  (3B) |
| Nan Wang  2019 | E: 50(35/15)  C: 50(38/12) | E: 22-49  C: 21-48 | E: Chushi Zhiyang ointment (q.d)  C: Tacrolimus ointment (q.d) | 4 weeks | ①⑤ | (1C) |
| Chunping Shen^*^  2017 | E: 98(58/40)  C: 97(56/41) | E: 1.02  C: 0.81 | E: Chushi Zhiyang ointment (b.i.d)  C: Hydrocortisone butyrate ointment (b.i.d) | 2 weeks | ①②⑤ | (1B) (2B) |
| Qin Ran  2017 | E: 56(36/20)  C: 58(36/22) | E: 0.41  C: 0.47 | E: Chushi Zhiyang ointment (b.i.d)  C: Dexamethasone cream (b.i.d) | 2 weeks | ①②⑤ | (1B) (2C) |
| Yangting Tang  2016 | E: 40(22/18)  C: 40(23/17) | E: 15-60  C: 17-59 | E: Chushi Zhiyang ointment (b.i.d)  C: Tacrolimus ointment (q.d) | 2 weeks | ①⑤ | (1C) |
| Aixing Huang  2015 | E: 50(30/20)  C: 50(30/20) | E: 0.33-10  C: 0.42-11 | E: Chushi Zhiyang ointment (b.i.d)  C: Triamcinolone acetonide and econazole ointment (b.i.d) | 3 weeks | ①⑤ | (1B) |
| Bing Li  2015 | E: 36(23/13)  C: 36(18/18) | E: 16-69  C: 17-70 | E: Chushi Zhiyang ointment (b.i.d)  C: Halometasone ointment (b.i.d) | 4 weeks | ①②③④⑤ | (1B) (2B) (3B) (4B) |
| Rong Ding  2014 | E: 60(31/29)  C: 60(28/32) | E: 18-70  C: 19-72 | E: Chushi Zhiyang ointment (b.i.d)  C: Desonide cream (b.i.d) | 4 weeks | ①⑤ | (1B) |
| Yue Shen  2012 | E: 28  C: 28 | E: 25-68  C: 25-68 | E: Chushi Zhiyang ointment (t.i.d)  C: Triamcinolone acetonide and econazole cream (b.i.d) | 4 weeks | ①⑤ | (1B) |

Supplementary Table 3: The characteristics of 144 randomized clinical trials on Chinese patent medicine for eczema (continued)

| First author | Sample size  (Male/Female) | Age Range or  Mean Age (year) | Intervention | Treatment  duration | Outcomes | Results |
| --- | --- | --- | --- | --- | --- | --- |
| Lin Geng  2010 | E: 36  C: 31 | E: 51.66  C: 52.17 | E: Chushi Zhiyang ointment (t.i.d)  C: Hydrocortisone butyrate cream (b.i.d) | 4 weeks | ①②⑤ | (1A) (2A) |
| Jianbo Zhang  2010 | E: 22(14/8)  C: 20(11/9) | E: 37.5  C: 36.9 | E: Chushi Zhiyang ointment (b.i.d)  C: Compound dexamethasone cream (b.i.d) | 3 weeks | ①⑤ | (1B) |
| Lirong Liu  2017 | E: 53(31/22)  C: 53(29/24) | E: 27-69  C: 27-68 | E: Chushi Zhiyang ointment (t.i.d to q.i.d) + Levocetirizine tablet (5mg/d)  C: Levocetirizine tablet (5mg/d) | 4 weeks | ①②③⑤ | (1A) (2A) (3A) |
| Jinmei Tao  2012 | E: 34(19/15)  C: 34(16/18) | E: NA  C: NA | E: Chushi Zhiyang ointment (b.i.d) + Epistatin capsule (10mg/d)  C: Desonide cream (b.i.d) + Epistatin capsule (10mg/d) | 18 days | ①⑤ | (1B) |
| Xiaoling Huang  2012 | E: 60(44/16)  C: 60(38/22) | E: 15-62  C: 16-65 | E: Chushi Zhiyang ointment (b.i.d) + Loratadine (10mg/d)  C: Halometasone cream (q.d) + Loratadine (10mg/d) | 4 weeks | ①⑤ | (1A) |
| Xiuhong Liu  2019 | E: 53(32/21)  C: 53(30/23) | E: 2-13  C: 1-14 | E: Chushi Zhiyang ointment (q.d) + Desonide cream (q.d) + Loratadine oral liquid (1.25-2.5mg/d)  C: Desonide cream (b.i.d) + Loratadine oral liquid (1.25-2.5mg/d) | 2 weeks | ①②⑤ | (1A) (2A) |
| Chengrui Li  2019 | E: 55(35/20)  C: 55(28/27) | E: 18-70  C: 19-72 | E: Chushi Zhiyang ointment (b.i.d) + Hydrocortisone butyrate cream (q.d)  C: Hydrocortisone butyrate cream (b.i.d) | 2 weeks | ① | (1A) |
| Yu Liu  2016 | E: 42  C: 42 | E: 38.9  C: 40.1 | E: Chushi Zhiyang ointment (b.i.d) + Triamcinolone acetonide and econazole cream  (q.d)  C: Triamcinolone acetonide and econazole cream (b.i.d) | 3 weeks | ①⑤ | (1A) |
| Jing Wei  2015 | E: 50(34/16)  C: 50(35/15) | E: 42-48  C: 40-46 | E: Chushi Zhiyang ointment (t.i.d to q.i.d) + Desonide cream (b.i.d to q.i.d)  C: Desonide cream (b.i.d to q.i.d) | 12 days | ①⑤ | (1A) |
| Cuifen Shen  2014 | E: 45  C: 45 | E: 0.25-3  C: 0.25-3 | E: Chushi Zhiyang ointment (b.i.d) + Desonide cream (b.i.d)  C: Desonide cream (b.i.d) | 2 weeks | ①⑤ | (1B) |
| Yinghu Zhang  2010 | E: 50(19/21)  C: 50(20/20) | E: 25-65  C: 15-65 | E: Chushi Zhiyang ointment (b.i.d) + Hydrocortisone butyrate ointment (b.i.d) +  Mizolastine tablet (10mg/d)  C: Hydrocortisone butyrate ointment (b.i.d) + Mizolastine tablet (10mg/d) | 5 days | ①⑤ | (1A) |
| Cuirong Deng  2009 | E: 43(24/19)  C: 43(23/20) | E: 0.22  C: 0.28 | E: Chushi Zhiyang ointment (b.i.d) + Hydrocortisone butyrate ointment (b.i.d)  C: Hydrocortisone butyrate ointment (b.i.d) | 2 weeks | ①②⑤ | (1A) (2A) |
| Xiaoyun Zhu  2014 | E: 34  C: 34 | E: 20-69  C: 20-69 | E: Binghuang Fule ointment (t.i.d) + Loratadine tablet (10mg/d)  C: Loratadine tablet (10mg/d) | 4 weeks | ① | (1A) |

Supplementary Table 3: The characteristics of 144 randomized clinical trials on Chinese patent medicine for eczema (continued)

| First author | Sample size  (Male/Female) | Age Range or  Mean Age (year) | Intervention | Treatment  duration | Outcomes | Results |
| --- | --- | --- | --- | --- | --- | --- |
| Zhiying Zhang  2014 | E: 31  C: 31 | E: 21-66  C: 21-66 | E: Binghuang Fule ointment (t.i.d) + Loratadine tablet (10mg/d)  C: Loratadine tablet (10mg/d) | 4 weeks | ①②⑤ | (1A) (2A) |
| Xueyan Liu  2013 | E: 90  C:90 | E: NA  C: NA | E: Binghuang Fule ointment (b.i.d) + Mizolastine (10mg/d)  C: Mizolastine tablet (10mg/d) | 2 weeks | ①⑤ | (1A) |
| Shi Wu  2012 | E: 50(27/23)  C: 50(26/24) | E: 21-65  C: 27-68 | E: Binghuang Fule ointment (b.i.d) + Loratadine tablet (10mg/d)  C: Loratadine tablet (10mg/d) | 4 weeks | ①③⑤ | (1A) (3A) |
| Yongzhong Chen  2012 | E: 55(34/21)  C: 55(32/23) | E:19-71  C:19-71 | E: Binghuang Fule ointment (b.i.d) + Ebastine tablet (10mg/d)  C: Halometasone cream (b.i.d) + Ebastine tablet (10mg/d) | 2 weeks | ①⑤ | (1B) |
| Hongjin Wang  2009 | E: 50(33/17)  C: 50(31/19) | E: 18-65  C: 18-65 | E: Binghuang Fule ointment (b.i.d) + Cetirizine tablet (10mg/d)  C: Hydrocortisone butyrate cream (b.i.d) + Cetirizine tablet (10mg/d) | 2 weeks | ①⑤ | (1B) |
| Xiangmei Liu  2014 | E: 43  C: 43 | E: 20-49  C: 20-49 | E: Binghuang Fule ointment (b.i.d to t.i.d)  C: Compound dexamethasone acetate cream (b.i.d to t.i.d) | 6 weeks | ① | (1A) |
| Zhengchao Liu  2014 | E: 60  C: 60 | E: 3-47  C: 3-47 | E: Binghuang Fule ointment (b.i.d to t.i.d)  C: Compound dexamethasone acetate cream (b.i.d to t.i.d) | 6 weeks | ① | (1A) |
| Li Xu  2012 | E: 40  C: 40 | E: NA  C: NA | E: Binghuang Fule ointment (b.i.d)  C: Hydrocortisone butyrate ointment (b.i.d) | 3 weeks | ①④ | (1B) (4B) |
| Yuangang Yang  2012 | E: 44  C: 45 | E: 15-73  C: 15-73 | E: Binghuang Fule ointment (b.i.d)  C: Compound flumetasone ointment (b.i.d) | 2 weeks | ①⑤ | (1C) |
| Cheng Li  2010 | E: 30  C: 30 | E: 22-71  C: 22-71 | E: Binghuang Fule ointment (b.i.d)  C: Mometasone furoate cream (b.i.d) | 4 weeks | ①⑤ | (1B) |
| Xueliang Zhao  2008 | E: 62(39/23)  C:62(37/25) | E:17-72  C:16-68 | E: Binghuang Fule ointment (b.i.d)  C: Triamcinolone acetonide ointment (b.i.d) | 1 week | ①②⑤ | (1B) (2B) |
| Zhenbang Zhang  2008 | E: 120(68/52)  C: 62(30/32) | E: 15-81  C: 16-78 | E: Binghuang Fule ointment (b.i.d)  C: Triamcinolone acetonide and econazole ointment (b.i.d) | 4 weeks | ①⑤ | (1B) |
| Wenguo Wei  2007 | E: 35(18/17)  C: 28(15/13) | E: 18-53  C: 19-55 | E: Binghuang Fule ointment (t.i.d)  C: Triamcinolone acetonide and econazole cream (b.i.d) | 2 weeks | ①⑤ | (1B) |
| Xiaojing Yang  2006 | E: 67(39/28)  C: 60(30/30) | E: 35.7  C: 34.3 | E: Binghuang Fule ointment (b.i.d)  C: Compound topical corticoid ointment (b.i.d) | 2-4 weeks | ①⑤ | (1B) |

Supplementary Table 3: The characteristics of 144 randomized clinical trials on Chinese patent medicine for eczema (continued)

| First author | Sample size  (Male/Female) | Age Range or  Mean Age (year) | Intervention | Treatment  duration | Outcomes | Results |
| --- | --- | --- | --- | --- | --- | --- |
| Shuping Liu  2009 | E: 100(58/42)  C:100(52/48) | E: 18-65  C: 15-64 | E: Binghuang Fule ointment (b.i.d) + Triamcinolone acetonide and miconazole nitrate  and neomycin sulfate cream (b.i.d)  C: Triamcinolone acetonide and miconazole nitrate and neomycin sulfate cream (q.i.d) | 4 weeks | ①④⑤ | (1A) (4B) |
| Fengju Xu  2015 | E:25(14/11)  C:24(19/5) | E: 18-62  C: 20-58 | E: Binghuang Fule ointment (b.i.d) + Levocetirizine capsule (10mg/d)  + Hydrocortisone butyrate cream (b.i.d)  C: Levocetirizine capsule (10mg/d) + Hydrocortisone butyrate cream (b.i.d) | 2 weeks | ①⑤ | (1A) |
| Jisheng Hu  2009 | E: 50(29/21)  C: 50(26/24) | E: 18-65  C: 15-64 | E: Binghuang Fule ointment (b.i.d) + Triamcinolone acetonide and econazole ointment  (b.i.d)  C: Triamcinolone acetonide and econazole ointment (b.i.d) | 2 weeks | ①⑤ | (1A) |
| Jiahui Xu  2006 | E: 30  C: 30 | E: 18-78  C: 18-78 | E: Binghuang Fule ointment (b.i.d) + Mometasone furoate cream (q.d)  C: Mometasone furoate cream (q.d) | 2-3 weeks | ①⑤ | (1A) |
| Weiquan Huang  2012 | E: 36(18/18)  C: 28(16/12) | E: 18-65  C:16-60 | E: Wudai ointment (b.i.d)  C: Mometasone furoate cream (b.i.d) | 3 weeks | ①⑤ | (1B) |
| Xiaojun Hu  2010 | E: 116  C: 121 | E: NA  C: NA | E: Wudai ointment (b.i.d to t.i.d)  C: Triamcinolone acetonide and econazole cream (b.i.d to t.i.d) | 4-8 weeks | ① | (1B) |
| Shunhua Zheng  2009 | E: 30  C: 25 | E: 7-81  C: 7-81 | E: Wudai ointment (b.i.d)  C: Triamcinolone acetonide and econazole cream (b.i.d) | 4 weeks | ①⑤ | (1B) |
| Wenhui Wang  2008 | E: 60(34/26)  C: 60(36/24) | E: 37.5  C: 36.9 | E: Wudai ointment (b.i.d)  C: Halometasone cream (b.i.d) | 3 weeks | ①⑤ | (1B) |
| Yang Liu  2011 | E: 50  C: 48 | E: 14-66  C: 14-66 | E: Wudai ointment (b.i.d) + Fexofenadine tablet (60mg/d)  C: Triamcinolone acetonide and econazole cream (b.i.d) + Fexofenadine tablet  (60mg/d) | 4 weeks | ①⑤ | (1B) |
| Hua Tian  2015 | E: 45  C: 45 | E: 20-65  C: 20-65 | E: Wudai ointment (b.i.d) + Mometasone furoate cream (b.i.d)  C: Mometasone furoate cream (b.i.d) | 4 weeks | ①⑤ | (1A) |
| Guiju Zhang  2012 | E: 30(18/12)  C: 30(16/14) | E: 44.17  C: 42.61 | E: Wudai ointment (q.d) + Mometasone furoate cream (q.d)  C: Mometasone furoate ointment (q.d) | 3 weeks | ② | (2A) |
| Shiwei Ding  2011 | E: 38(28/10)  C: 36(28/8) | E: 29-46  C: 28-46 | E: Wudai ointment (b.i.d) + Clobetasol propionate cream (b.i.d)  C: Clobetasol propionate cream (b.i.d) | 2 weeks | ①⑤ | (1A) |
| Xuesong Li  2014 | E: 37(13/24)  C: 36(11/25) | E: 26-55  C: 25-54 | E: Paeonol ointment (t.i.d)  C: Triamcinolone acetonide and econazole cream (t.i.d) | 4 weeks | ①②③⑤ | (1B) (2B)  (3C) |

Supplementary Table 3: The characteristics of 144 randomized clinical trials on Chinese patent medicine for eczema (continued)

| First author | Sample size  (Male/Female) | Age Range or  Mean Age (year) | Intervention | Treatment  duration | Outcomes | Results |
| --- | --- | --- | --- | --- | --- | --- |
| Zhengjuan Liang  2015 | E: 54  C: 54 | E: 0.17-2  C: 0.17-2 | E: Paeonol ointment (b.i.d) + Hydrocortisone butyrate cream (b.i.d)  C: Hydrocortisone butyrate cream (b.i.d) | 1 week | ① | (1A) |
| Zhongyou Wang  2013 | E: 53  C: 52 | E: 0-2  C: 0-2 | E: Paeonol ointment (b.i.d) + Hydrocortisone butyrate cream (b.i.d)  C: Hydrocortisone butyrate cream (b.i.d) | 1 week | ①⑤ | (1A) |
| Jinhua Liu  2010 | E: 33  C: 33 | E: NA  C: NA | E: Paeonol ointment (b.i.d) + Halometasone cream (b.i.d)  C: Halometasone cream (b.i.d) | 1 week | ①⑤ | (1A) |
| Wubian Wei  2007 | E: 44  C: 43 | E: 0-1  C: 0-1 | E: Paeonol ointment (q.d) + Hydrocortisone butyrate ointment (q.d)  C: Hydrocortisone butyrate ointment (q.d) | 1 week | ① | (1B) |
| Linhua Bao  2010 | E: 46(28/18)  C: 34(18/16) | E: 18-49  C: 18-50 | E: Paeonol ointment (q.d) + Clobetasol propionate cream (q.d) (Week 1 and Week 2)  Paeonol ointment (b.i.d) (Week 3 and Week 4)  C: Clobetasol propionate cream (b.i.d) (Week 1, Week 2, Week 3 and Week 4) | 4 weeks | ①②⑤ | (1B) (2B) |
| Qi Qi  2009 | E: 43  C: 43 | E:18-70  C:18-70 | E: Paeonol ointment (t.i.d) + Dexamethasone liniment (q.d)  C: Dexamethasone liniment (b.i.d) | 2 weeks | ① | (1B) |
| Yanping Bai  2007 | E: 30 (11/19)  C: 30 (9/21) | E: 35.72  C: 37.50 | E: Pifukang lotion (t.i.d) + Loratadine tablet (10mg/d)  C: Normal saline (t.i.d) + Loratadine tablet (10mg/d) | 4 days | ①②③⑤ | (1A) (2A)  (3A) |
| Lan Wu  2004 | E: 100 (58/42)  C: 60 (26/34) | E: 0.83-82  C: 1.5-73 | E: Pifukang lotion (b.i.d)  C: Triamcinolone acetonide and econazole cream (b.i.d) | 1 week | ①⑤ | (1B) |
| Baotian Yu  1999 | E: 30 (9/21)  C: 25 (12/13) | E: 16-74  C: 16-70 | E: Pifukang lotion (b.i.d)  C: Fluocinolone acetonide cream (b.i.d) | 1 week | ①②⑤ | (1B) (2B) |
| Minying Li  1997 | E: 70  C: 30 | E: 0.08-65  C: 0.08-65 | E: Pifukang lotion (b.i.d)  C: Fluocinolone acetonide cream (b.i.d) | 1 week | ① | (1A) |
| Yongshan Li  2014 | E: 60(28/32)  C: 60(30/30) | E: 12-78  C: 10-82 | E: Pifukang lotion (b.i.d) + Triamcinolone acetonide and econazole cream (b.i.d)  C: Triamcinolone acetonide and econazole cream (b.i.d) | 2 weeks | ①⑤ | (1A) |
| Jian Mo  2007 | E: 45  C: 45 | E: NA  C: NA | E: Pifukang lotion (b.i.d) + Hydrocortisone butyrate ointment (b.i.d)  C: Hydrocortisone butyrate ointment (b.i.d) | 1 week | ①⑤ | (1A) |
| Dingquan Yang  2005 | E: 43  C: 40 | E: 53.8  C: 49.2 | E: Pifukang lotion (t.i.d) + Mometasone furoate cream (q.d)  C: Mometasone furoate cream (q.d) | 2-4 weeks | ①②③ | (1A) (2A)  (3A) |
| Xiaoying Yuan  2013 | E: 58(32/26)  C: 47(25/22) | E: 37-65  C: 38-67 | E: Sophora alopecuroide oil liniment (b.i.d)  C: Triamcinolone acetonide and econazole cream (b.i.d) | 2 weeks | ①⑤ | (1B) |

Supplementary Table 3: The characteristics of 144 randomized clinical trials on Chinese patent medicine for eczema (continued)

| First author | Sample size  (Male/Female) | Age Range or  Mean Age (year) | Intervention | Treatment  duration | Outcomes | Results |
| --- | --- | --- | --- | --- | --- | --- |
| Xiaoying Yuan  2013 | E: 62(32/30)  C: 54(30/24) | E: 35.3  C: 36.1 | E: Sophora alopecuroide oil liniment (b.i.d)  C: Triamcinolone acetonide and econazole cream (b.i.d) | 2 weeks | ①⑤ | (1B) |
| Xiaoying Yuan  2013 | E: 119  C: 88 | E: 6-70  C: 6-70 | E: Sophora alopecuroide oil liniment (b.i.d)  C: Triamcinolone acetonide and econazole cream (b.i.d) | 4 weeks | ①⑤ | (1B) |
| Xiaoying Yuan  2013 | E: 73  C: 68 | E: 19-60  C: 18-60 | E: Sophora alopecuroide oil liniment (b.i.d)  C: Triamcinolone acetonide and econazole cream (b.i.d) | 2 weeks | ①⑤ | (1B) |
| Kefeng Gu  2008 | E: 50(30/20)  C: 50(33/27) | E: 12-52  C: 15-68 | E: Sophora alopecuroide oil liniment (b.i.d)  C: Hydrocortisone butyrate ointment (b.i.d) | 3 weeks | ①⑤ | (1B) |
| Surong Da  2011 | E: 50(28/22)  C: 39(23/16) | E: 18-60  C: 21-59 | E: Sophora alopecuroide oil liniment (b.i.d) + Levocetirizine tablet (10mg/d)  C: Hydrocortisone butyrate cream (b.i.d) + Levocetirizine tablet (10mg/d) | 2 weeks | ①⑤ | (1B) |
| Junjie Qian  2006 | E: 62  C: 61 | E: 0-3  C: 0-3 | E: Erfukang liniment (b.i.d)  C: Hydrocortisone butyrate ointment (b.i.d) | 1 week | ① | (1B) |
| Hongmei Li  2005 | E: 106  C: 50 | E: 0-2  C: 0-2 | E: Erfukang liniment (q.d to b.i.d)  C: Triamcinolone acetonide and econazole cream (b.i.d) | 2 weeks | ①⑤ | (1B) |
| Wenfeng Geng  2011 | E: 60  C: 32 | E: NA  C: NA | E: Erfukang liniment (t.i.d) + Loratadine syrup (2.5-5mg/d)  C: Loratadine syrup (2.5-5mg/d) | 4 weeks | ① | (1A) |
| Xiaolin Rao  2012 | E: 45(19/26)  C: 45(18/27) | E: 0.08-1.67  C: 0.08-1.58 | E: Erfukang liniment (b.i.d) + Hydrocortisone butyrate cream (b.i.d)  C: Hydrocortisone butyrate cream (b.i.d) | 1 week | ①⑤ | (1A) |
| Jianhui Wang  2010 | E: 120(62/58)  C: 120(54/66) | E: 0.5-2  C: 0.5-2 | E: Erfukang liniment (t.i.d) + Desonide cream (q.d)  C: Desonide cream (q.d) | 1 week | ①②⑤ | (1A) (2A) |
| Weibin Hu  2009 | E: 80  C: 50 | E: 0.17-2  C: 0.17-2 | E: Erfukang liniment (b.i.d) + Hydrocortisone butyrate ointment (b.i.d)  C: Hydrocortisone butyrate ointment (b.i.d) | 5 days | ①⑤ | (1A) |
| Yine Hu  2015 | E: 30(20/10)  C: 30(18/12) | E: 22-63  C: 20-58 | E: Qiangyue cream (b.i.d)  C: Compound dexamethasone acetate cream (b.i.d) | 4 weeks | ①②③⑤ | (1A) (2A)  (3A) |
| Lifu Zhang  2015 | E: 62  C: 61 | E: 0.14-2  C: 0.14-2 | E: Qiangyue cream (b.i.d)  C: Hydrocortisone butyrate ointment (b.i.d) | 2 weeks | ①⑤ | (1B) |
| Weiguo Sun  2009 | E: 40(22/18)  C: 40(24/16) | E: 15-63  C: 16-65 | E: Qiangyue cream (b.i.d)  C: Halometasone cream (b.i.d) | 2 weeks | ①③⑤ | (1B) (3A) |
| Xu Zhang  2007 | E: 40(24/16)  C: 40(21/19) | E: 16-61  C: 18-63 | E: Qiangyue cream (b.i.d)  C: Halometasone cream (b.i.d) | 2 weeks | ①③⑤ | (1B) (3A) |

Supplementary Table 3: The characteristics of 144 randomized clinical trials on Chinese patent medicine for eczema (continued)

| First author | Sample size  (Male/Female) | Age Range or  Mean Age (year) | Intervention | Treatment  duration | Outcomes | Results |
| --- | --- | --- | --- | --- | --- | --- |
| Houliang Lou  2015 | E: 65(40/25)  C: 65(38/27) | E: 0.25-12  C: 0.19-12 | E: Qiangyue cream (b.i.d to t.i.d) + Loratadine syrup (2.5-5ml/d)  C: Loratadine syrup (2.5-5ml/d) | 2 weeks | ①④⑤ | (1A) (4A) |
| Weiguo Sun  2009 | E: 46(26/20)  C: 34(16/18) | E: 0.25-5  C: 0.67-5 | E: Qiangyue cream (q.d) + Hydrocortisone butyrate cream (q.d) + Loratadine syrup  (5-10ml/d) (Week 1 and Week 2)  Qiangyue cream (b.i.d) + Loratadine syrup (5-10ml/d) (Week 3 and Week 4)  C: Hydrocortisone butyrate cream (q.d) + Loratadine syrup (5-10ml/d)  (Week 1, Week 2, Week 3 and Week 4) | 4 weeks | ①②⑤ | (1B) (2B) |
| Jijun Lyu  2010 | E: 47(6/41)  C: 46(3/43) | E:18-51  C:17-50 | E: Meilu Xiaocuo ointment (b.i.d) + Mizolastine tablet (10mg/d)  C: Mizolastine tablet (10mg/d) | 2 weeks | ①⑤ | (1A) |
| Aiming Shu  2009 | E: 59  C: 50 | E:24.3  C:24.3 | E: Meilu Xiaocuo ointment (t.i.d)  C: Hydrocortisone butyrate cream (b.i.d) | 2 weeks | ①⑤ | (1A) |
| Xiaodan Luo  2018 | E: 48(22/26)  C: 46(24/22) | E: 1-8  C: 1-6.8 | E: Geranium ointment (b.i.d) + Desonide cream (b.i.d) + Levocetirizine oral liquid  (5-10ml/d)  C: Desonide cream (b.i.d) + Levocetirizine oral liquid (5-10ml/d) | 2 weeks | ①⑤ | (1A) |
| Minghong Yu  2013 | E: 66  C: 66 | E: 15-60  C: 15-60 | E: Mayinglong musk hemorrhoid cream (b.i.d)  C: Triamcinolone acetonide and urea cream (b.i.d) | 2 weeks | ①⑤ | (1A) |
| Dan Du  2014 | E: 63(35/28)  C: 64(37/27) | E: 2-50  C: 2-50 | E: Chushi Zhiyang lotion (t.i.d to q.i.d) + Desonide cream (b.i.d to q.i.d)  C: Desonide cream (b.i.d to q.i.d) | 12 days | ① | (1A) |
| Minfang Yang  2011 | E: 43(22/21)  C: 43(23/20) | E: 16-75  C: 16-75 | E: Shuangzishen lotion (b.i.d) + Triamcinolone acetonide and urea ointment (b.i.d)  + Epinastine capsule (20mg/d) + Cyproheptadine tablet (2mg/d)  C: Triamcinolone acetonide and urea ointment (b.i.d) + Epinastine capsule (20mg/d)  + Cyproheptadine tablet (2mg/d) | 3 weeks | ①⑤ | (1A) |
| Zhao Yu  1996 | E: 75  C: 68 | E: NA  C: NA | E: Jieeryin lotion (b.i.d)  C: Triamcinolone acetonide and urea ointment (b.i.d) | 1-2 weeks | ① | (1A) |
| Hui Gao  2011 | E: 40(23/17)  C: 35(19/16) | E: NA  C: NA | E: Xiaofeng Zhiyang granule (90g/d)  C: Loratadine tablet (10mg/d) | 4 weeks | ①⑤ | (1A) |
| Yun Jiang  2016 | E: 63(29/34)  C: 63(31/32) | E: 18-56  C: 21-54 | E: Xiaofeng Zhiyang granule (36g/d) + Compound flumetasone ointment (b.i.d)  C: Compound flumetasone ointment (b.i.d) | 4 weeks | ①⑤ | (1A) |

Supplementary Table 3: The characteristics of 144 randomized clinical trials on Chinese patent medicine for eczema (continued)

| First author | Sample size  (Male/Female) | Age Range or  Mean Age (year) | Intervention | Treatment  duration | Outcomes | Results |
| --- | --- | --- | --- | --- | --- | --- |
| Caiyun Liu  2015 | E: 40(18/22)  C: 40(15/25) | E: 18-65  C: 18-65 | E: Xiaofeng Zhiyang granule (36g/d) + Ebastine tablet (10mg/d) + Hydrocortisone  butyrate ointment (q.d to b.i.d)  C: Ebastine tablet (10mg/d) + Hydrocortisone butyrate ointment (q.d to b.i.d) | 4 weeks | ①②③⑤ | (1A) (2A)  (3A) |
| Xin Wang  2013 | E: 50  C: 50 | E: 15-50  C: 20-45 | E: Xiaofeng Zhiyang granule (36g/d) + Cetirizine tablets (10mg/d)  C: Cetirizine tablets (10mg/d) | 4 weeks | ①⑤ | (1A) |
| Wangui Cai  2010 | E: 60(30/30)  C: 60(30/30) | E: 17-52  C: 22-57 | E: Xiaofeng Zhiyang granule (10.5g/d) + Triamcinolone acetonide and econazole  cream (t.i.d)  C: Triamcinolone acetonide and econazole cream (t.i.d) | 8 weeks | ①②⑤ | (1A) (2A) |
| Rong Zhang  2019 | E: 48  C: 49 | E: NA  C: NA | E: Piminxiao capsule (4.8g/d) + Cetirizine tablet (20mg/d)  C: Cetirizine tablet (20mg/d) | 4 weeks | ①③⑤ | (1A) (3A) |
| Dongbao Su  2013 | E: 74  C: 58 | E: 16-50  C: 16-50 | E: Piminxiao capsule (4.8g/d) + Levocetirizine tablet (5mg/d)  C: Levocetirizine tablet (5mg/d) | 2 weeks | ① | (1A) |
| Junxiang Lyu  2017 | E: 18(12/6)  C: 18(13/5) | E: 18-56  C: 18-55 | E: Baixuanxiatare tablet (3.6g/d)  C: Cetirizine tablet (10mg/d) | 2 weeks | ① | (1A) |
| Peijiu Wang  2011 | E: 31  C: 31 | E: NA  C: NA | E: Baixuanxiatare tablet (3.6g/d) + Cetirizine tablet (10mg/d) + Triamcinolone  acetonide and urea cream (b.i.d)  C: Cetirizine tablet (10mg/d) + Triamcinolone acetonide and urea cream (b.i.d) | 2 weeks | ①⑤ | (1A) |
| Jing Bai  2008 | E: 90  C: 90 | E: 18-63  C: 18-63 | E: Phellodendron bark capsule (9g/d)  C: Terfenadine tablet (120mg/d) | 2-4 weeks | ①⑤ | (1A) |
| Xu Zhang  2003 | E: 60  C: 60 | E: 18-64  C: 18-64 | E: Phellodendron bark capsule (9g/d)  C: Terfenadine tablet (120mg/d) | 2-4 weeks | ①②⑤ | (1A) (2A) |
| Ting Zhao^*^  2015 | E: 71(31/40)  C: 36(19/17) | E: 38.26  C: 39.48 | E: Fangfeng Tongsheng granule (6g/d)  C: Simulated agent granule (6g/d) | 2 weeks | ①②⑤ | (1A) (2A) |
| Yang Qiu  2018 | E: 31(15/16)  C: 32(14/18) | E: 41.59  C: 40.73 | E: Sophora flavescens capsule (3.96-5.94g/d) + Cetirizine tablet (10mg/d)  C: Cetirizine tablet (10mg/d) | 1 week | ① | (1A) |

E: Experimental group; C: Control group; NA: Not available;

^*^ a multiple-center randomized controlled trial; ^#^ an article published in English

①: Total effective rate; ②: Overall score of disease severity; ③: visual analogue scale for pruritus; ④: recurrent rate; ⑤:adverse effects;

(1A): The total effective rate of the experimental group at the end of treatment is significantly higher than that of the control group;

(1B): The total effective rates between two groups at the end of treatment are not statistically different;

(1C): The total effective rate of the experimental group at the end of treatment is significantly lower than that of the control group;

(2A): The overall score of disease severity of the experimental group at the end of treatment is significantly lower than that of the control group;

(2B): The overall scores of disease severity between two groups at the end of treatment are not statistically different;

(2C): The overall score of disease severity of the experimental group at the end of treatment is significantly higher than that of the control group;

(3A): The visual analogue scale for pruritus of the experimental group at the end of treatment is significantly lower than that of the control group;

(3B): The visual analogue scales for pruritus between two groups at the end of treatment are not statistically different;

(3C): The visual analogue scale for pruritus of the experimental group at the end of treatment is significantly higher than that of the control group;

(4A): The recurrent rate of the experimental group at the end of follow-up period is significantly lower than that of the control group;

(4B): The recurrent rates between two groups at the end of follow-up period are not statistically different.
